# Immunophenotyping of Acute Inflammatory Exacerbations of Lung Injury Driven by Mutant Surfactant Protein-C: A Role for Inflammatory Eosinophils

**DOI:** 10.3389/fphar.2022.875887

**Published:** 2022-04-27

**Authors:** Jacklyn Nguyen, Brittnie S. Armstrong, Sophie Cowman, Yaniv Tomer, Shivakumar R. Veerabhadraiah, Michael F. Beers, Alessandro Venosa

**Affiliations:** ^1^ Department of Pharmacology and Toxicology, University of Utah, Salt Lake City, UT, United States; ^2^ Pulmonary, Allergy, and Critical Care Division, Department of Medicine, Perelman School of Medicine, University of Pennsylvania, Philadelphia, PA, United States; ^3^ PENN-CHOP Lung Biology Institute, University of Pennsylvania, Philadelphia, PA, United States

**Keywords:** eosinophil, pulmonary fibrosis (PF), inflammatory exacerbations, corticosteroid, surfactant protein C (SP-C), GATA1

## Abstract

Acute inflammatory exacerbations (AIEs) represent immune-driven deteriorations of many chronic lung conditions, including COPD, asthma, and pulmonary fibrosis (PF). The first line of therapy is represented by broad-spectrum immunomodulation. Among the several inflammatory populations mobilizing during AIEs, eosinophils have been identified as promising indicators of an active inflammatory exacerbation. To better study the eosinophil-parenchymal crosstalk during AIE-PF, this work leverages a clinically relevant model of inflammatory exacerbations triggered by inducible expression of a mutation in the alveolar epithelial type 2 cell Surfactant Protein-C gene [SP-C^I73T^]. Unbiased single-cell sequencing analysis of controls and SP-C^I73T^ mutants at a time coordinated with peak eosinophilia (14 days) defined heightened inflammatory activation, chemotaxis, and survival signaling (IL-6, IL-4/13, STAT3, Glucocorticoid Receptor, mTOR, and MYC) in eosinophils. To study the impact of eosinophils in inflammatory exacerbations, the SP-C^I73T^ line was crossed with eosinophil lineage deficient mice (GATA1^Δdbl^) to produce the SP-C^I73T^GATA1^KO^ line. Time course analysis (7–42 days) demonstrated improved lung histology, survival, and reduced inflammation in SP-C^I73T^GATA1^KO^ cohorts. Spectral flow cytometry of tissue digests confirmed eosinophil depletion in GATA1^KO^ mice and the absence of a compensatory shift in neutrophils and immature monocyte recruitment. Eosinophil deletion resulted in progressive monocyte-derived macrophage accumulation (14 days post-injury), combined with declines in CD3^+^CD4^+^ lymphocyte and B220^+^ B cell abundance. Histochemical analysis revealed atypical inflammatory cell activation in SP-C^I73T^GATA1^KO^ mice, with reduced numbers of Arg-1^+^ and iNOS^+^ cells, but increases in *tgfb1* mRNA expression in bronchoalveolar lavage cells and tissue. Dexamethasone treatment (1 mg/kg daily, i.p.) was utilized to investigate corticosteroid efficacy in highly eosinophilic exacerbations induced by mutant SP-C^I73T^. Dexamethasone successfully reduced total and eosinophil (CD11b^+^SigF^+^CD11c^−^) counts at 14 days and was linked to reduced evidence of structural damage and perivascular infiltrate. Together, these results illustrate the deleterious role of eosinophils in inflammatory events preceding lung fibrosis and demonstrate the efficacy of corticosteroid treatment in highly eosinophilic exacerbations induced by mutant SP-C^I73T^.

## Introduction

The lung parenchyma displays a remarkable capacity to maintain homeostasis and manage stress (either in the form of infection or toxicant exposure) through an organized and temporally-defined inflammatory response. This is achieved through continuous communication between epithelial cells and resident (alveolar and interstitial macrophages) or recruited (monocyte, eosinophils, neutrophils) effector cells of the immune compartment. Disruption of this balance influences inflammatory and repair mechanisms, that may tip the balance in favor of pathologies characteristic of chronic parenchymal lung disease (Ganguly et al., 2011). Mutation of key functional genes (i.e., telomeres, mucins, pulmonary surfactants) have been shown to produce such aberrant phenotype, and have been linked with several chronic pulmonary diseases, including pulmonary fibrosis (PF) (Crossno et al., 2010; Juarez et al., 2015; Povedano et al., 2015; Collard et al., 2016; Hambly et al., 2017; Leuschner and Behr, 2017).

To model this, we leveraged a recently characterized murine model expressing a clinical PF-linked mutation of the alveolar epithelial-restricted surfactant protein C gene, the isoleucine-to-threonine missense substitution at position 73 (“SP-C^I73T^”). Our group has previously characterized a viable murine strain which, as a result of induction of the mutant SP-C^I73T^ isoform, generates a complex multicellular inflammatory exacerbation of lung injury progressing to fibrosis (Brasch et al., 2004; Tsakiri et al., 2007; Abou Taam et al., 2009; Nureki et al., 2018). In particular, the SP-C mutant induced exacerbation produces sequential waves of monocytosis and neutrophilia, leading to eosinophilia at the apex of the inflammatory response (Venosa et al., 2019). This is dissimilar to established models of lung fibrosis such as nitrogen mustard, bleomycin, radiation, or silica, which pioneered the dynamics of mononuclear myeloid populations in the fibrotic injury (Venosa et al., 2016; Misharin et al., 2017; Meziani et al., 2018; Hou et al., 2019; Mukherjee et al., 2021).

This is significant since the epidemiological analysis of inflammatory exacerbations of PF, asthma, and COPD consistently identifies a subset of individuals producing non-canonical inflammation, dominated by polymorphonucleocytes (Kakugawa et al., 2016; Kerkhof et al., 2017; Nakagome and Nagata, 2018). Insufficient understanding of the exact events accompanying acute inflammatory exacerbations of pulmonary fibrosis is primarily responsible for the paucity of targeted and effective therapies clinically available, as demonstrated by decades of unsuccessful clinical trials (e.g., PANTHER, ASCEND, INPULSIS, CleanUP) (Raghu et al., 2012; Richeldi et al., 2014; Martinez et al., 2021). Indeed, current recommendations by the American Thoracic Society, the European Respiratory Society, the Japanese Respiratory Society, and the Latin American Thoracic Association (ATS/ERS/JRS/ALAT) on the management of acute inflammatory exacerbations of PF include supportive care and largely ineffective broad spectrum immunomodulation (corticosteroids), alone or in combination with antioxidants (N-acetylcysteine), cytotoxic drugs (azathioprine, cyclophosphamide), and antibacterial drugs (azithromycin) (Juarez et al., 2015).

The lung epithelium is essential to harmonize the mixed cell infiltrate (in terms of origin, phenotypic activation, and maturation state) of an acute exacerbation episode, which combined spatially heterogeneous injury to asynchronous inflammation (Cameron et al., 2005; Abou Taam et al., 2009; Crossno et al., 2010). Largely observational evidence links disproportionate mobilization of peripheral monocytes and their persistence in the lung as monocyte-derived alveolar macrophages with poor outcome (Misharin et al., 2017; Reyfman et al., 2019; Scott et al., 2019; Adams et al., 2020). Proof of concept studies from our group and others have determined that depletion of resident and infiltrating (Ly6C^+^ and CCR2^+^) mononuclear myeloid cells significantly impacts the trajectory of fibrogenic injury, yet these results may have limited therapeutic potential due to their influx early in the exacerbation process, prior to any structural damage (Young et al., 2016; Venosa et al., 2019). Similarly, disease endotypes characterized by neutrophilic exacerbations have been identified as particularly refractory to corticosteroid therapy, and thus linked to poor patient prognosis (Wark and Gibson, 2006). By comparison, there is more sporadic evidence documenting bronchoalveolar lavage and blood eosinophilia in a subset of PF individuals, suggesting they could serve as potential predictors of disease outcome for some patients (Libby, 1987; Yousem, 2000; Brix et al., 2016; Kakugawa et al., 2016). Beyond these observations in PF, eosinophils’ function remains ambivalent in lung health, with evidence demonstrating their protective role in helminth infections as well as deleterious function during inflammatory exacerbations of asthma and COPD (Huang and Appleton, 2016; Nakagome and Nagata, 2018; Yun et al., 2018). By using the SP-C mutant model, which consistently produces eosinophil-enriched inflammatory exacerbations leading to a fibrotic phenotype, this work allows us to interrogate the role of eosinophil in the fibrogenic injury. At the same time, it allows us to test whether corticosteroid efficacy in PF and other chronic respiratory diseases may, at least in part, conditional on eosinophilia (Cheng, 2018; Barnes, 2019; Chipps et al., 2020).

Here we identify the phenotype of infiltrating eosinophils accumulating during SP-C^I73T^-induced inflammatory exacerbation, examine the impact of eosinophils deletion on the progression of fibrosis, and confirm the benefits of corticosteroid therapy in eosinophilic exacerbations. Single-cell RNA-sequencing of lung tissue identifies 9 clusters of cells at the peak of SP-C^I73T^ induced inflammation. Eosinophils produced a pro-survival (MYC, YAP, AKT, and mTOR signaling) and highly inflammatory transcriptome (IL-6 and STAT3). Deletion of eosinophil lineage (GATA1 knock out) was linked to reduced inflammation and tissue injury. Furthermore, pharmacological intervention using dexamethasone was effective in blunting eosinophilia and reducing SP-C^I73T^-induced injury. Together, these data highlight the deleterious role of eosinophils in PF and provide supporting evidence for the use of corticosteroid therapy in eosinophilic exacerbations.

## Materials and Methods

### Reagents

Tamoxifen (non-pharmaceutical grade) was purchased from Sigma-Aldrich (St Louis, MO). Dexamethasone was purchased from VetOne through MXI Animal Health (Boise, ID). Giemsa cytological stain was purchased from Sigma-Aldrich. Antibodies used for *in situ* hybridization, immunohistochemical and flow cytometric analysis were: *tgfb1* (Cat # 407751, Advanced Cell Diagnostics, ACD); Arg-1 (Cat # ab91279; 1:1500, Abcam, Waltham, MA); iNOS (Cat # ab15323; 1:200, Abcam); CD103 (Cat # ab224202; 1/750; Abcam); CCR2 (Cat # ab273050; 1:400, Abcam); CD206 (Cat # EPR20844; 1:750, Cell Signaling Technology, Danvers, MA); Eosinophil Peroxidase (Cat # sc-19148; 1:250, Santa Cruz Biotechnology, Inc., Dallas, TX); CD16/32 (clone 93; eBiosciences, San Diego, CA), CD11c (clone # N418; APC, Biolegend); Ly6G (clone # 1A8; APC/Fire 750, Biolegend); CD5 (clone # 53-7.3; AF700, Biolegend); Ly6C (clone # HK1.4; PerCP/Cy5.5, Biolegend); CX_3_CR1 (clone # SA011F11; AF488, Biolegend); CD45 (clone # 30-F11; BUV395, BD Biosciences); CD11b (clone # M1/70; BV510, Biolegend); CD3 (clone # 17A2; BV711, Biolegend); CD206 (clone # C068C2; BV785, Biolegend); CD64 (clone # X54-5/7.1; BUV605, Biolegend); CD170/SigF (clone # S17007L; B421, Biolegend); CD4 (clone # RM4-5; BV570, Biolegend); CD192/CCR2 (clone # SA203G11; PE, Biolegend); CD43 (clone # S11; PE/Cy7, Biolegend); NK1.1/CD161 (clone # PK136; PE/Cy5, Biolegend); CD103 (clone # 2E7; PE/Dazzle594, Biolegend); CD24 (clone # M1/69; BUV496, BD Biosciences; Franklin Lakes, NJ); CD45R/B220 (clone # RA3-6B2; BUV737, BD Biosciences); CD8a (clone # 53-6.7; BUV805, BD Biosciences); Fixable Viability Stain (Cat # 566332; 440UV; BD Biosciences). All other reagents were purchased from Thermo Fisher Scientific, Inc. (Waltham, MA).

### Murine Model of SP-C^I73T^ Induced Lung Injury

All animal studies were performed in accordance with the revised ARRIVE guidelines (Percie Du Sert et al., 2020). Tamoxifen inducible SP-C^I73T^ mice were generated as previously reported (9). Briefly, the SP-C^I73T^ founder line (expressing a Neomycin cassette) was crossed with a mouse line expressing an estrogen receptor (ER)-2 controlled Flp-O recombinase strain knocked into the Rosa26 locus (Jackson Laboratory, Bar Harbor, ME) to generate the inducible-SP-C^I73T^Flp line. Adult homozygote SP-C^I73T^Flp mice received tamoxifen (175 mg/kg in corn oil, oral gavage) at 8–12 weeks of age. Both male and female animals were used for the studies. Time points for these studies (7 days, 14 days, and 42 days) were selected to investigate initiation, peak injury, and fibrotic remodeling following an acute inflammatory exacerbation episode. Control mice are represented as pooled data from tamoxifen treated SP-C^I73T^ not expressing Flp-O recombinase or oil (vehicle) treated Flp-O expressing SP-C^I73T^ mice. In studies using dexamethasone, daily intraperitoneal injections (1 mg/kg) were performed starting on day 5 or 7 up to day 14. Controls received sterile saline solution. To delete the eosinophil lineage, SP-C^I73T^ mice were then crossed to homozygosity with a GATA1 line (Stock No.: 005653, Jackson Laboratories). The resulting triple transgenic line was backcrossed to C57BL/6J for 10 generations before it was used for these studies. All mice were housed under pathogen-free conditions in AALAC approved barrier facilities at the Perelman School of Medicine (University of Pennsylvania), and Skaggs College of Pharmacy, University of Utah. All experiments were approved by the Institutional Animal Care and Use Committee at the University of Utah and the University of Pennsylvania.

### Lung Histology, Histochemistry, and *In Situ* Hybridization

Whole lungs were fixed by tracheal instillation of 10% neutral buffered formalin at constant pressure (25 cm H_2_O). Following paraffin embedding, 6 μm sections were cut and stained with Hematoxylin & Eosin (H&E) by the Associated Regional and University Pathologists Inc., at the University of Utah. The selection of representative histological specimens within this manuscript was based on semiquantitative scoring (0–5). The final score was the product of a combination of individual scores for inflammatory cell influx, presence of edema, size, and number of injury foci (septal remodeling) (adapted from (Matute-Bello et al., 2008). Scores were produced by two observers blinded to the genotype and experimental conditions.

Immunostaining of deparaffinized tissue sections was performed as previously described (Venosa et al., 2015). Briefly, after antigen retrieval using citrate buffer (10.2 mM sodium citrate, pH 6.0, for 20 min) and quenching of endogenous peroxidase with 3% hydrogen peroxide in methanol (30 min), non-specific binding was blocked with PBS + 10% serum according to primary antibody origin. For studies involving immunocytochemistry, initial xylenes/alcohol processing, antigen retrieval, and citrate retrieval were not required. Freshly spun cytospins of BAL were initially fixed in chilled (−20°C) acetone and dried overnight. Non-specific binding was blocked with PBS + 5% serum according to primary antibody origin + 0.15% triton-X for 45 min. Appropriate serum/IgG controls, each diluted in blocking buffer, were applied for overnight incubation at 4°C in a humidified chamber. Following incubation with the biotinylated secondary antisera (Vectastain Elite ABC kit, Vector Labs, Burlingame, CA) for 30 min (room temperature), staining was visualized using a Peroxidase Substrate Kit DAB (Vector Labs) and counterstained with Harris Modified Hematoxylin (Thermo Fisher Scientific, Inc.). Slides were dehydrated in alcohol gradient (50%–100%), followed by xylenes. Coverslipped slides were allowed to dry overnight before image acquisition. For quantification of immunohistochemistry, 5 foci of injury from each specimen were imaged at ×400 magnification, and positively staining cells enumerated. Counts from each specimen were then averaged and displayed as a single data point. Three distinct animals per study group were utilized for quantification.

In other studies, *in situ* hybridization was performed before immunohistochemical staining. Briefly, paraffin-embedded sections were deparaffinized in xylene and 100% EtOH. This was followed by peroxidase quenching in H_2_O_2_ (10 min, away from light), antigen retrieval (RNAscope Target Retrieval Reagent, ACD), and protease IV treatment (RNAscope Protease IV Reagent, ACD). A *Tgfb1* ACD probe was then incubated for 2 h in a hybridization oven (40°C), a step followed by a series of signal amplification steps and chromogenic development as indicated by manufacturer protocol. Slides were then washed and the immunohistochemistry blocking step resumed as described above. Image acquisition occurred the following day to allow the mounting reagent to fully dry. Images (×400 magnification) were acquired using a Zeiss Axioscope 7 (Carl Zeiss Meditec, Inc., Dublin, CA) and equally processed using Adobe Photoshop (San Jose, CA).

### Blood and Bronchoalveolar Lavage Fluid (BALF) Analysis

Before lung lavage, 100 μL of blood was collected from the posterior vena cava and red blood cells immediately lysed in ACK buffer (Thermo Fisher Scientific, Inc.), spun down at 400 × *g* for 6 min, and resuspended in 1 ml of 0.9% sodium chloride saline solution. Subsequently, BALF was collected from mice using five sequential lavages of 1 ml sterile saline and processed for analysis as previously described (9). Briefly, cell pellets obtained by centrifuging BALF samples at 400 × *g* for 6 min were re-suspended in 1 ml of saline solution. Both blood and BALF were enumerated using a NucleoCounter (New Brunswick Scientific, Edison, NJ). BAL cytospins containing approximately 10^4^ cells were stained with Giemsa for 20 min for preliminary immunological assessment.

### Flow Cytometry and Cell Sorting for Identification of Immune Populations

Following BALF collection, lungs were cleared of blood by cardiac perfusion with saline solution, removed from the chest cavity, minced, and transferred into a 50 ml conical tube and incubated (37°C, 30 min) in DMEM + 5% FBS + 2 mg/ml Collagenase D (Cat #11088866001, Roche, Indianapolis, IN). Digested lungs were passed through 70-μm nylon mesh strainer to obtain a single-cell suspension, counted, and mixed with ACK Lysis Buffer (Thermo Fisher Scientific) to remove any remaining red blood cells. BALF and tissue cell pellet (1 × 10^6^ cells) were resuspended in 100 μL staining buffer (PBS+0.1% sodium azide) and incubated with anti-mouse CD16/32 antibody for 10 min at 4°C to block nonspecific binding. This was followed by 30 min incubation with fluorescently-tagged antibodies or appropriate isotype controls (0.25–1.5 μg/10^6^ cells, 4°C). Cells were then spun and resuspended in staining buffer for viability staining (30 min at 4°C). Cells were fixed in 2% paraformaldehyde and analyzed with a Cytek Aurora spectral analyzer (Cytek Biosciences, Fremont, CA). All populations were identified following forward and side scatter selection of singlet CD45^+^ viable cells. Cells were sequentially gated to identify resident alveolar macrophages (SigF^+^CD11b^−^CD11c^+^), eosinophils (SigF^int^CD11b^+^CD11c^−^), neutrophils (Ly6G^+^), NK cells (CD11b^int^NK1.1^+^), B cells (B220^+^), dendritic cells (CD4^+^CD103^+^ and CD8^+^CD103^+^) and lymphocytes (CD3^+^CD4^+^ and CD3^+^CD8^+^). All analysis was performed using FlowJo software (FlowJo LLC, Ashland, Oregon).

### Single-Cell RNA Sequencing Tissue Preparation and Analysis

In one experiment, single-cell suspensions obtained through Collagenase D digestion underwent RNA single-cell sequencing (sc-RNA) at the University of Utah Huntsman Cancer Institute High-Throughput Genomics Shared Resource core. The 10x Genomics Chromium single-cell gene expression platform (10x Genomics, Pleasanton, CA) was used to assess the gene expression of single cells. The libraries were prepared according to the 10x Genomics Single Cell 3′ Gene expression Library prep protocol V3, then analyzed using Agilent D1000 ScreenTape on an Agilent Technology 2200 TapeStation system (Agilent Technologies, Santa Clara, CA) and quantified by PCR using KAPA Biosystems Library Quantification Kit for Illumina Platforms (KAPA Biosystems, Roche, Branchburg NJ). Libraries were sequenced on a NovaSeq 6000 (Illumina, San Diego, CA) with 2 × 150 bp paired-end reads. 10x Genomics Cell Ranger was used to process raw sequencing data. Further quality control processing was conducted with the Seurat (4.0.4) package for R (4.1.1) where genes detected in less than three cells were removed, and only cells with a minimum of 200 detected genes were taken for downstream analysis. Cells with >10% mitochondrial gene expression were removed. Dimension reduction and clustering were performed using the Seurat (4.0.4) package. Clusters were identified using common gene markers and examining differential gene expression results. Further pathway analysis was conducted using Ingenuity Pathway Analysis (Qiagen, Germantown, MD). Raw data were deposited in NCBI’s Gene Expression Omnibus (Edgar et al., 2002) and are accessible through GEO Series accession number GSE196657 (https://www.ncbi.nlm.nih.gov/geo/query/acc.cgi?acc=GSE196657).

### Statistics

All data are presented with dot-plots and group mean ± SEM unless otherwise indicated. Statistical analyses were performed with Prism GraphPad 9.0 (GraphPad Software, San Diego, CA). Student’s t-tests were used for paired data; for analyses involving multiple groups, one-way or two-way analysis of variance (ANOVA) was performed with post hoc testing as indicated. Histopathological assessment was shown as median with interquartile range, and Kruskal-Wallis test performed. Survival analyses were performed using Log Rank (Mantel-Cox) test. In all cases, statistical significance was considered at *p* ≤ 0.05. With the exception of single cell sequencing and immunohistochemical analysis, for which staining was performed on a single batch to avoid interexperimental variability, all other experiments shown (survival studies, BAL analysis, dexamethasone treatment, flow cytometry) are shown as the combined results of at least three separate experiments.

## Results

### Single-Cell Sequencing Analysis of SP-C^I73T^ Lungs at the Peak Acute Inflammatory Exacerbation

**FIGURE 1:**
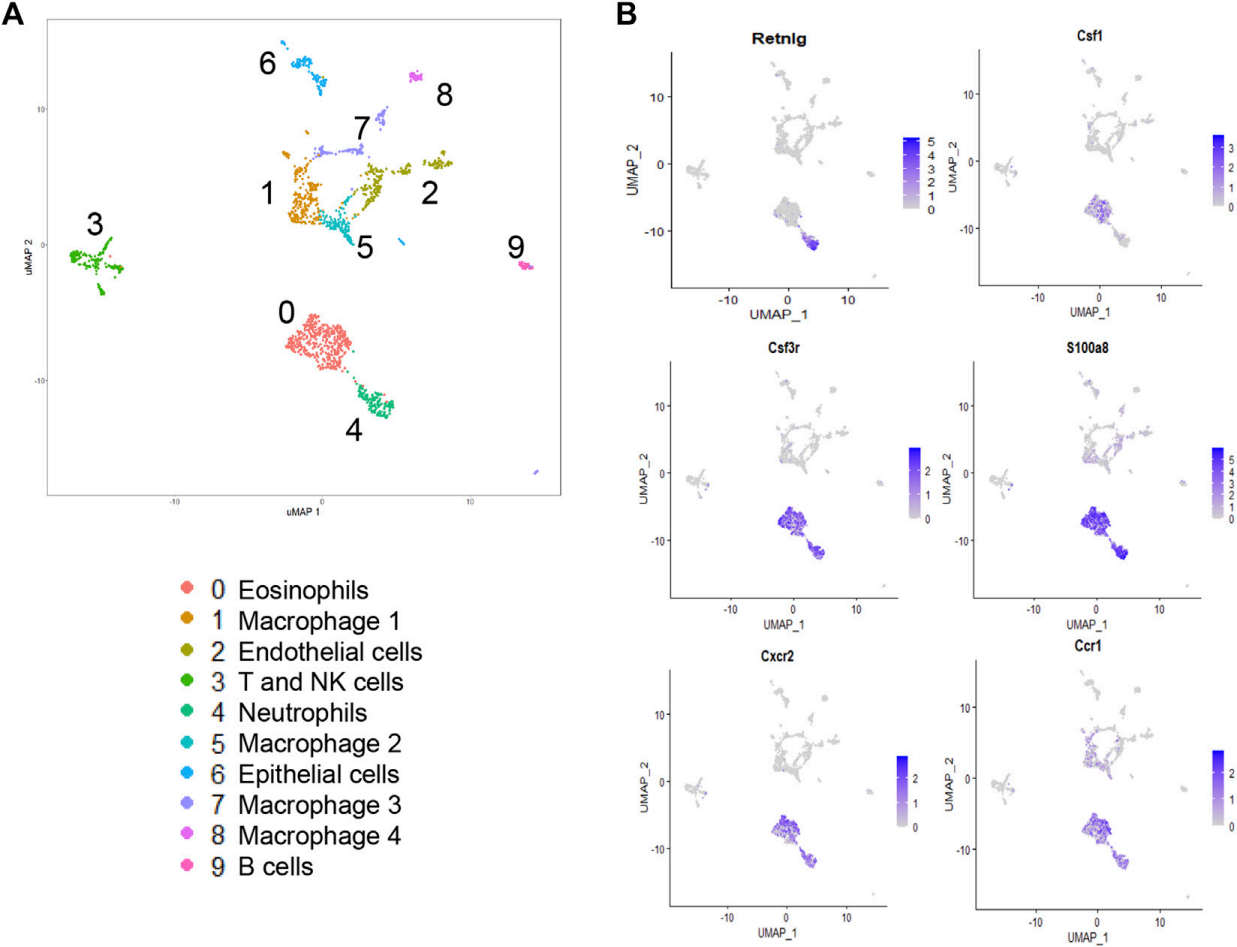
Single-cell RNA sequencing analysis of lung tissue digests during SP-C^I73T^ induced acute inflammatory exacerbations. Single cell sequencing analysis was performed on collagenase digested single cell suspensions from SP-C^I73T^ mice 14 days after injury. **(A)** Umap of the nine cell populations clustered using the Seurat analysis package. **(B)** Umaps with overlay of gene expression expressed solely by neutrophils (*retnlg*), eosinophils (*csf1*), or both but at levels higher than all other cluster (*csf3*, *s100a9*, *cxcr2*, *ccr1*).

**FIGURE 2:**
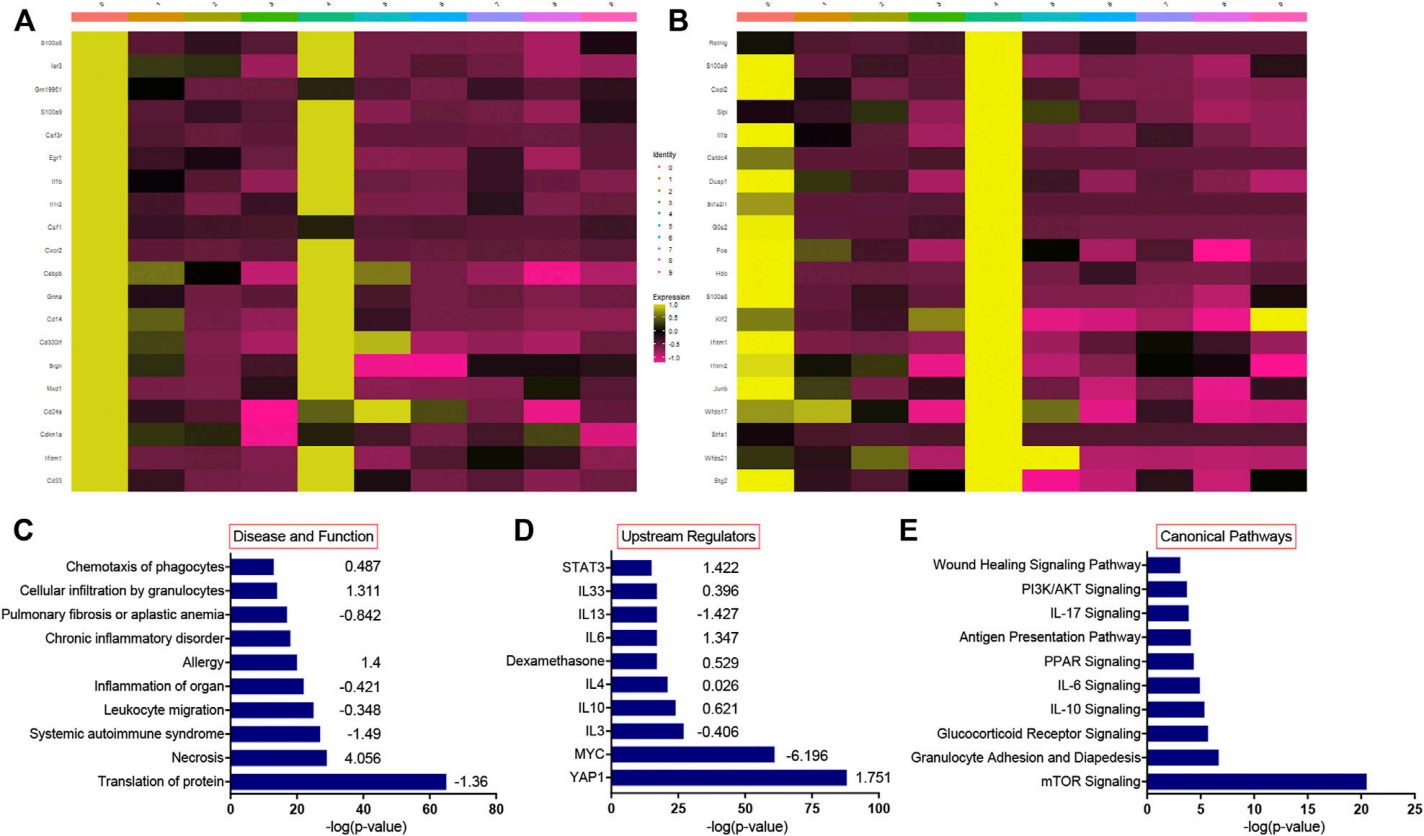
Single-cell RNA sequencing pathway analysis during SP-C^I73T^ induced acute inflammatory exacerbations. Single cell RNA-sequencing analysis was performed on collagenase digested single cell suspensions from control (CTL, oil treated SP-C^I73T^ mice) or SP-C^I73T^ mice 14 days after injury. **(A,B)** Heat maps of the top 20 most expressed genes in the eosinophil and neutrophil cluster relative to all other clusters. **(C–E)** Ingenuity pathway analysis was performed for the differentially expressed genes of the eosinophil population. Graph represents 10 activated pathways with respect to **(C)** disease and function, **(D)** upstream regulators, and **(E)** canonical pathways are presented with their corresponding −log(p-value) (x-axis) and predicted directionality.

We have previously shown that lung injury generated by induction of mutant SP-C^I73T^ expression is accompanied by multicellular inflammation, peaking at 14 days, when maximal eosinophil influx was observed [27]. We, therefore, performed transcriptional profiling of these populations through unbiased single-cell sequencing analysis. After quality control steps (See *Methods*), dimensionality reduction and clustering were performed using the Seurat package (4.0.1), leading to the identification of nine distinct cell clusters. We identified eosinophils and neutrophils, as well as lymphocytes (*Cd3*, *Nkg7*), B cells (*Ighd*), endothelial (*Pecam1*), epithelial cells (*Scgb3a2*, *Sftpb*), as well as four distinct populations of macrophages (Figure 1A; Supplementary Figure S1). The transcriptomic pattern of cluster 0 and cluster 4 (polymorphonucleated cells) were defined by expression of *Itgam, Csf3*, *S100a8/9*, *Cxcr2*, *Ccr1*, *Trem1*, *Lcn2*, and *Cd14* (Figures 2A,B), with neutrophil-eosinophil distinction resulting from distinct expression of *Retnlg* and *Csf1*, respectively (Figure 1B; Supplementary Figure S1). Ingenuity Pathway Analysis (IPA) of the differentially expressed genes in the eosinophil cluster identified activation of pathways related to chemotaxis and mobility, inflammatory responses, and protein translation (Figure 2C). Upstream regulator and canonical pathway analysis identified shared engagement of signals linked to inflammatory interleukins (IL-4, IL-13, IL-3, IL-33, IL-6) and survival/differentiation (YAP1, MYC, mTOR), as well as dexamethasone and glucocorticoid responses (Figures 2D,E).

### Impact of Eosinophil Lineage Deletion on SP-C^I73T^ Induced Injury and Inflammatory Cell Dynamics

**FIGURE 3:**
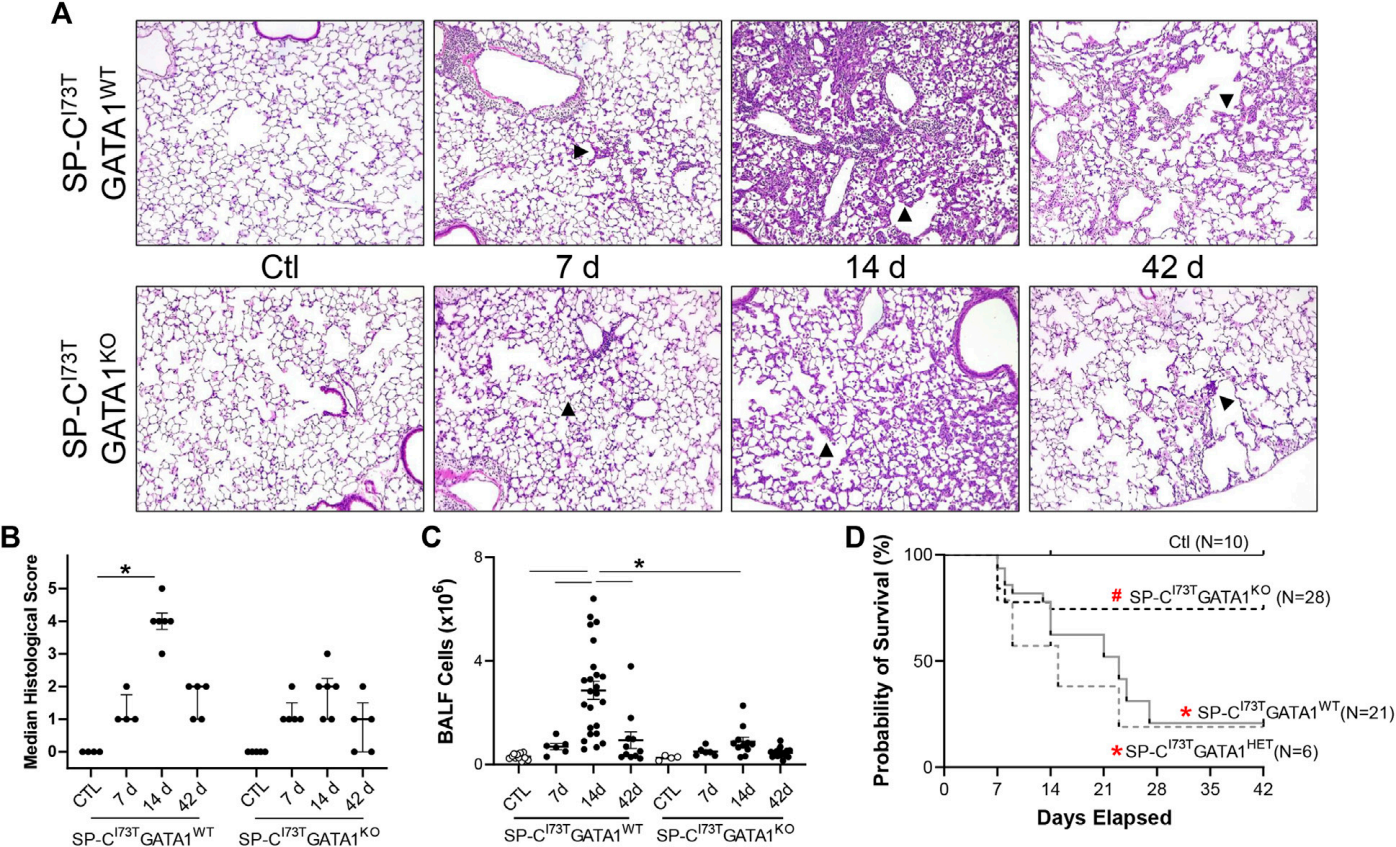
Effects of genetic eosinophil lineage ablation on lung injury, survival and inflammation following SP-C^I73T^ mutant induced injury. **(A,B)** Hematoxylin and Eosin stain and histological score of tissue sections from oil treated controls (Ctl), or tamoxifen treated SP-C^I73T^GATA1^WT^ and SP-C^I73T^GATA1^KO^ mice at 7 days, 14 days, or 42 days post injury. Arrowheads represent regions of interest, including septal remodeling edema, and cell infiltrate. Original magnification: ×100. Representative images from at least 3 mice/group are shown. Scoring, shown as Median ± interquartile range, was based on weighted examination of tissue remodeling, inflammation, size and number of foci of injury. **(C)** Bronchoalveolar lavage fluid (BALF) cell counts from oil treated controls (Ctl), or tamoxifen treated SP-C^I73T^GATA1^WT^, SP-C^I73T^GATA1^HET^, and SP-C^I73T^GATA1^KO^ mice at 7 days, 14 days, or 42 days post injury. Data are presented as mean ± SEM (*n* = 4–23 mice/group), analyzed using two-way ANOVA. A *p* < 0.05 (*) was considered significant. Lines mark significant groups. **(D)** Kaplan–Meier survival analysis from oil treated controls (Ctl), or tamoxifen treated SP-C^I73T^GATA1^WT^, SP-C^I73T^GATA1^HET^, and SP-C^I73T^GATA1^KO^ mice up to 42 days post injury. Analysis include mice found dead or displaying body weight loss equating >20% of experiment initiation. *p < 0.05 compared to control mice; ^#^ *p* < 0.05 compared to SP-C^I73T^GATA1^WT^ and SP-C^I73T^GATA1^HET^ mice by Log-Sum (Mantel-Cox) Rank test.

Guided by previous evidence that inflammatory eosinophils represent the predominant peripheral population in the lung at the peak of inflammatory exacerbations (Nureki et al., 2018), we opted to cross SP-C^I73T^ mice with ΔdblGATA1 mice, a line characterized by complete ablation of the eosinophil lineage (referred to as SP-C^I73T^GATA1^KO^). Histopathological analysis of control SP-C^I73T^GATA1^WT^ and SP-C^I73T^GATA1^KO^ lungs did not highlight significant changes. Similarly, there was no significant difference observed 7 days post mutant induction, characterized by perivascular edema in infiltrate. By 14 days, at the peak of inflammation, we noted significant injury only in the SP-C^I73T^GATA1^WT^, with a less conspicuous injury score in the eosinophil depleted cohorts (Figures 3A,B). By 42 days, as the inflammatory injury dissipated, the histological analysis did not reveal a significant difference between the two strains. By comparison, deletion of eosinophil lineage curbed collagen accumulation, as shown by picrosirius red staining 14 days and 42 days post induction (Supplementary Figures S2A–C). Similarly, there was a robust reduction in bronchoalveolar lavage (BAL) counts in SP-C^I73T^GATA1^KO^ mice (Figure 3C). Unsurprisingly, this response was matched with reduced mortality at the peak of inflammatory exacerbation (55% SP-C^I73T^GATA1^WT^ and GATA1^HET^ vs. 12.7% SP-C^I73T^GATA1^KO^, at 14 days) (Figure 3D).

**FIGURE 4:**
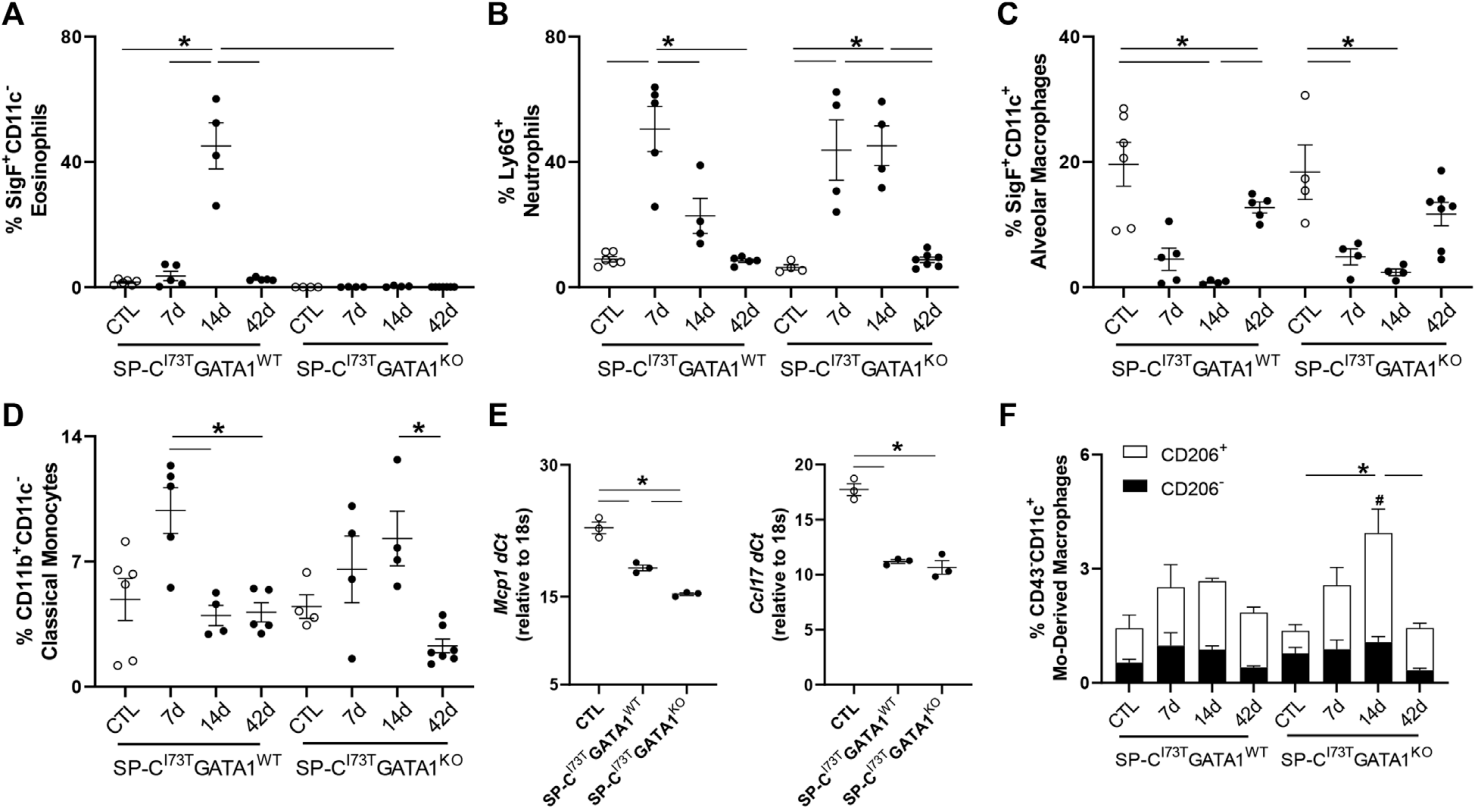
Effects of genetic eosinophil lineage ablation on myeloid inflammatory cell dynamics following SP-C^I73T^ induced injury. **(A–D,F)** Flow cytometric quantification of **(A)** Ly6G^+^ neutrophils, **(B)** SigF^+^CD11c^−^CD11b^+^ eosinophils, **(C)** SigF^+^CD11c^+^CD11b^−^ alveolar macrophages, **(D)** CD11b^+^CD11c^−^ classical monocytes, and **(F)** CD43^−^CD11c^+^ monocyte-derived macrophages prepared from collagenase digested lungs from oil treated controls (Ctl), or tamoxifen treated SP-C^I73T^GATA1^WT^ and SP-C^I73T^GATA1^KO^ mice at 7 days, 14 days, or 42 days post injury. A *p* < 0.05 (*) was considered significant. Lines mark significant groups. **(E)** mRNA analysis of BAL cells isolated from oil treated controls (Ctl), or tamoxifen treated SP-C^I73T^GATA1^WT^ and SP-C^I73T^GATA1^KO^ mice 14 days post injury. Data is expressed as Delta-Ct relative to 18s housekeeping gene. A *p* < 0.05 (*) was considered significant. Lines mark significant groups. **(F)** Relative abundance of total CD43^−^CD11c^+^ monocyte-derived macrophages (MoDMs) in SP-C^I73T^GATA1^WT^ and SP-C^I73T^GATA1^KO^ mice. Data are presented as mean ± SEM (*n* = 5–8 mice/group), analyzed using two-way ANOVA. A *p* < 0.05 was considered significant. Note that each column comprises CD206^−^ (black); and CD206^+^ (white) subsets. *Identifies significant differences in the total population compared to air controls. #Identifies significant differences within CD206 subsets versus control groups. Data are presented as mean ± SEM (*n* = 5–8 mice/group), analyzed using two-way ANOVA.

**FIGURE 5:**
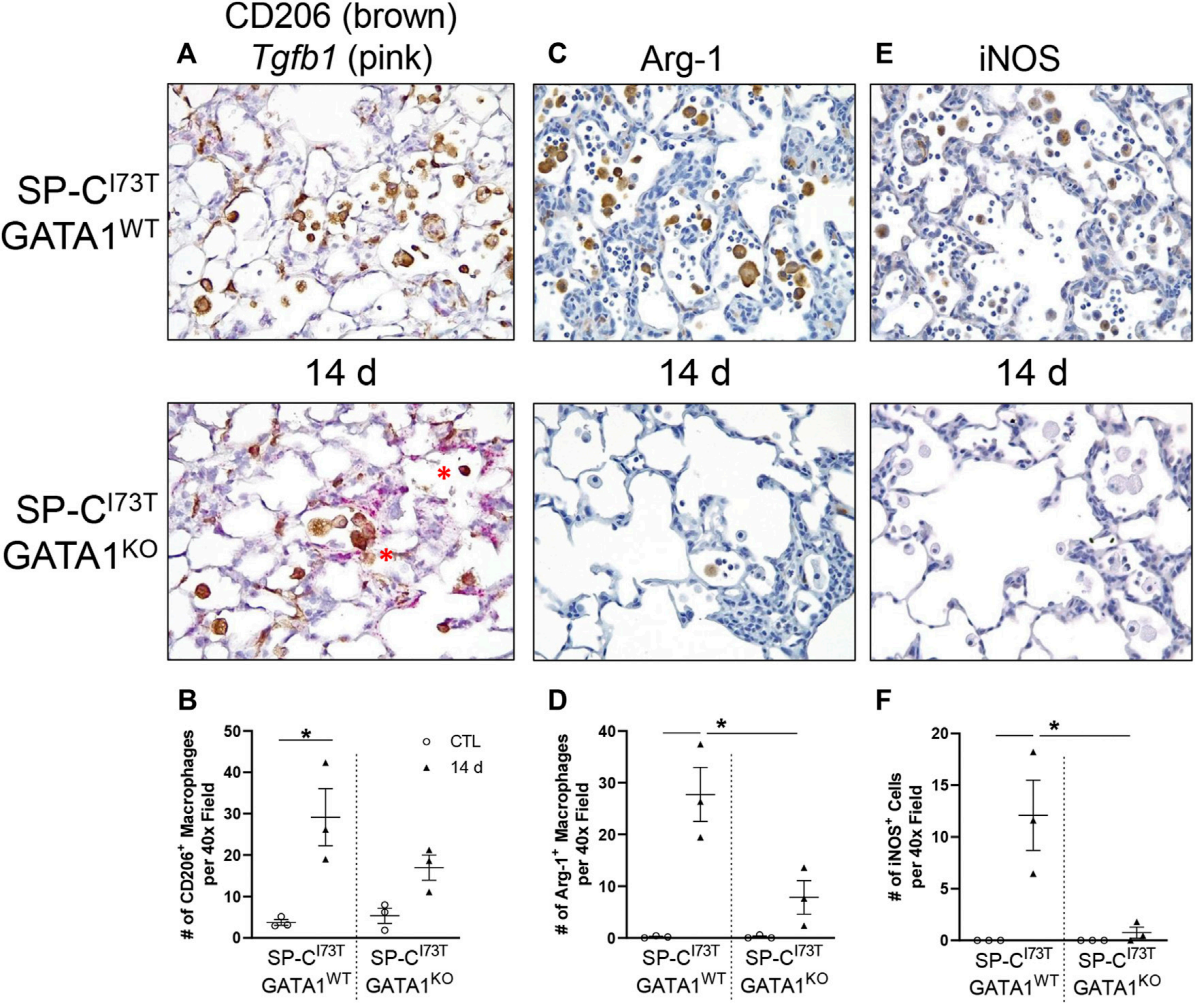
Effects of genetic eosinophil lineage ablation on pro-inflammatory activation following SP-C^I73T^ induced injury. Histochemical analysis alone or in combination with *in situ* hybridization of SP-C^I73T^GATA1^WT^ and SP-C^I73T^GATA1^KO^ lungs 14 days post SP-C^I73T^ induced injury. Sections were immunostained with antibody to **(A)** CD206 + *Tgfb1* (*in situ* hybridization). mRNA visualization is shown in pink. Protein expression was visualized using a DAB Vectastain kit (brown). Arrowheads indicate cells expressing the receptor. **(C)** Arg-1 or **(E)** iNOS. Binding was visualized using a Vectastain kit. Original magnification, ×400. Representative sections from three mice/group are shown. Quantification of each respective antibody is shown in **(B,D,F)** as the average number of brown/positive inflammatory cells in ×400 images of regions of injury. Data are presented as mean ± SD (*n* = 3 mice/group), analyzed using two-way ANOVA. A *p* < 0.05 (*) was considered significant.

Flow cytometric analysis of digested lung tissues is presented in both relative abundance and absolute counts, due to significant differences in inflammatory cell influx between the two mouse cohorts. Comprehensive summary tables are included (Tables 1–4). Initial analysis ascertained complete ablation of SigF^+^CD11c^−^ eosinophils in SP-C^I73T^GATA1^KO^ mice (Figure 4A) and prolonged neutrophilia up to 14 days in SP-C^I73T^GATA1^KO^ mice (Figure 4B). Analysis of mononuclear myeloid cells showed no changes in SigF^+^CD11c^+^ alveolar macrophage abundance over the 42 days period under investigation (Figure 4C). By comparison, SP-C induced injury promoted transient CD11b^+^ monocytosis in SP-C^I73T^GATA1^WT^ mice 7 days post-injury, with SP-C^I73T^GATA1^KO^ mice displaying persistent accumulation up to 14 days (Figure 4D). Notably, the expression for the monocyte chemokine ligand *Mcp1/ccl2* in BAL cells displayed as delta Ct relative to housekeeping expression was significantly increased in SP-C^I73T^GATA1^WT^ mice at 14 days. This response was amplified in SP-C^I73T^GATA1^KO^ cohorts (Figure 4E). By comparison, levels for the non-canonical monocyte/macrophage recruitment chemokine *Ccl17* and the eosinophil recruitment chemokine *Il5* were comparable between the two groups (Figure 4E and not shown). Within the myeloid compartment, we also noted increases in the relative abundance of CD11b^+^CD43^−^CD11c^+^ monocyte-derived macrophages (MoDMs), in particular, a subset expressing the CD206/mannose receptor, in SP-C^I73T^GATA1^KO^ mice 14 days post-injury (Figure 4F). When relative abundances were back-calculated to absolute counts, accumulation of CD11b^+^CD43^−^CD11c^+^ MoDMs increased more than 10-fold in SP-C^I73T^GATA1^WT^ mice, a response blunted by eosinophil-deletion (Supplementary Figure S3A) Histochemical analysis of CD206^+^ cells in lung tissue at 14 days revealed increased clustering of these cells within areas of tissue remodeling in eosinophil-competent and–deficient mice (Figures 5A,B, asterisks). Interestingly, *in situ* hybridization analysis showed *tgfb1* mRNA expression in the lung parenchyma in proximity to CD206^+^ macrophage clusters, exclusively in SP-C^I73T^GATA1^KO^ mice. Due to the heterogeneity of injury observed during an exacerbation, and the impact of eosinophil deletion on injury foci size at the intersection of inflammation and remodeling, *tgfb1* quantification did not lead to consistent results. Additional histochemical analysis of macrophage activation revealed heightened expression of both Arg-1 and iNOS in SP-C^I73T^GATA1^WT^ mice, but not in the GATA1^KO^ lungs (Figures 5C–F). No substantial expression of any of these markers was noted in control tissues (Supplementary Figures S4A–C).

**TABLE 1:** Myeloid populations (absolute counts) in the lung of SP-C^I73T^GATA1^WT^ and SP-C^I73T^GATA1^KO^ mice.

Myeloid	Genotype	CTL *(n=6)*	7d *(n=5)*	14d *(n=4)*	42d *(n=4)*
Alveolar Macrophages CD11b^-^SigF^+^CD11c^+^	SPC^I73T^GATA1^WT^	259,935 ± 69,337	69,497 ± 18,748^a^	51,011 ± 7,521^a^	88,647 ± 38,581^a^
	SPC^I73T^GATA1^KO^	298,884 ± 40,855	115,992 ± 25,619	102,316 ± 54,449^a^	64,501 ± 11,706^a^
Neutrophils Ly6G^+^	SPC^I73T^GATA1^WT^	195,170 ± 55,515	1,154,877 ± 293,241^a^	1,450,980 ± 283,112^a^	71,535 ± 21,498^b^ ^,^ ^c^
	SPC^I73T^GATA1^KO^	130,783 ± 50,233	1,281,653 ± 434,042^a^	1,450,949 ± 309,120^a^	54,829 ± 14,936^b^ ^,^ ^c^
Eosinophils CD11b^+^SigF^+^CD11c^-^	SPC^I73T^GATA1^WT^	45,123 ± 19,459	94,665 ± 48,537	3,653,455 ± 1,656,062^a^ ^,^ ^b^	21,337 ± 6,945^c^
	SPC^I73T^GATA1^KO^	473.5 ± 133.3	2,428 ± 567.1	9,250 ± 4,036	570.9 ± 108.0
Infiltrating Cells CD11b^+^	SPC^I73T^GATA1^WT^	335,649 ± 105,182	387,439 ± 94,093	1,150,500 ± 408,208^a^ ^,^ ^b^	191,425 ± 62,767^c^
	SPC^I73T^GATA1^KO^	319,110 ± 105,971	423,493 ± 102,750	815,239 ± 260,128	138,001 ± 22,392
Classical Monocytes CD43^-^CD11c^-^	SPC^I73T^GATA1^WT^	130,163 ± 46,341	240,565 ± 59,288	266,850 ± 55,701	38,392 ± 15,823
	SPC^I73T^GATA1^KO^	119,852 ± 54,176	198,776 ± 68,247	328,923 ± 194,314	13,905 ± 3,425^c^
Classical Inflammatory Monocytes CD43^-^CD11c^-^Ly6Chi	SPC^I73T^GATA1^WT^	103,449 ± 36,945	136,572 ± 62,353	169,549 ± 29,264	30,986 ± 13,093
	SPC^I73T^GATA1^KO^	94,641 ± 44,420	160,302 ± 54,909	209,704 ± 120,108	8,407 ± 2,408
Interstitial Macrophages CD43^+^CD11c^+^	SPC^I73T^GATA1^WT^	79,343 ± 25,757	61,260 ± 20,304	31,713 ± 3,375	48,485 ± 19,428
	SPC^I73T^GATA1^KO^	122,329 ± 45,077	45,021 ± 16,400	69,169 ± 48,037	28,414 ± 4,739
Alternative Monocytes CD43^+^CD11c^-^	SPC^I73T^GATA1^WT^	15,421 ± 4,665	10,811 ± 3,843	3,288 ± 801	13,280 ± 5,264
	SPC^I73T^GATA1^KO^	8,211 ± 2,554	11,165 ± 3,803	17,628 ± 14,825	4,403 ± 962
Monocyte-derived Macrophages CD43^-^CD11c^+^	SPC^I73T^GATA1^WT^	110,676 ± 48,063	128,884 ± 46,965	848,648 ± 361,581^a^ ^,^ ^b^	91,267 ± 32,827^c^
	SPC^I73T^GATA1^KO^	68,757 ± 10,487	168,713 ± 43,567	399,518 ± 28,162	91,279 ± 17,009
Monocyte-derived Macrophages CD206^-^CD43^-^CD11c^+^	SPC^I73T^GATA1^WT^	38,269 ± 13,386	49,774 ± 17,034	273,404 ± 118,480^a^ ^,^ ^b^	18,383 ± 5,923^c^
	SPC^I73T^GATA1^KO^	40,002 ± 9,122	56,707 ± 12,376	114,235 ± 20,437	20,245 ± 4,028
Monocyte-derived Macrophages CD206^+^CD43^-^CD11c^+^	SPC^I73T^GATA1^WT^	72,407 ± 36,154	79,110 ± 29,988	575,244 ± 243,824^a^ ^,^ ^b^	72,885 ± 27,499^c^
	SPC^I73T^GATA1^KO^	28,754 ± 8,466	112,006 ± 32,105	285,283 ± 38,006	71,034 ± 13,272

Single cell suspensions of SP-C^I73T^GATA1^WT^ and SP-C^I73T^GATA1^KO^ lung digests was assessed by flow cytometry, enriched in viable CD45^+^ singlets, and analyzed following gating strategy described in the *Methods* section. Absolute counts are presented as means ± SEM (N = 4-6 mice/condition). Data were analyzed by two-way ANOVA, with Tukey post-hoc test.

a Significant compared to controls (CTL).

b Significant compared to 7 d group.

c Significant compared to 14 d group.

**TABLE 2:** Myeloid populations (relative abundance) in the lung of SP-C^I73T^GATA1^WT^ and SP-C^I73T^GATA1^KO^ mice.

Myeloid	Genotype	CTL *(n=6)*	7d *(n=5)*	14d *(n=4)*	42d *(n=4)*
Alveolar Macrophages CD11b^-^SigF^+^CD11c^+^	SPC^I73T^GATA1^WT^	19.63 ± 3.50	4.47 ± 1.78^a^	0.81 ± 0.15^a^	12.73 ± 0.88^c^
	SPC^I73T^GATA1^KO^	18.38 ± 4.34	4.84 ± 1.27^a^	2.38 ± 0.54^a^	11.68 ± 1.87
Neutrophils Ly6G^+^	SPC^I73T^GATA1^WT^	8.94 ± 0.82	50.52 ± 7.20^a^	22.77 ± 5.58^b^	8.45 ± 0.57^b^
	SPC^I73T^GATA1^KO^	6.32 ± 0.84	43.80 ± 9.62^a^	45.17 ± 6.31^a^	8.74 ± 0.89^b^ ^,^ ^c^
Eosinophils CD11b^+^SigF^+^CD11c^-^	SPC^I73T^GATA1^WT^	1.63 ± 0.34	3.58 ± 1.50	45.13 ± 7.39^a^ ^,^ ^b^	2.53 ± 0.21^c^
	SPC^I73T^GATA1^KO^	0.03 ± 0.003	0.10 ± 0.02	0.28 ± 0.11	0.10 ± 0.01
Infiltrating Cells CD11b^+^	SPC^I73T^GATA1^WT^	13.73 ± 2.24	17.65 ± 1.98	15.39 ± 0.74	21.33 ± 0.86
	SPC^I73T^GATA1^KO^	16.03 ± 1.44	14.82 ± 2.09	23.61 ± 2.47	24.30 ± 2.06^b^
Classical Monocytes CD43^-^CD11c^-^	SPC^I73T^GATA1^WT^	4.88 ± 1.17	9.65 ± 1.45	3.98 ± 0.57^b^	4.16 ± 0.54^c^
	SPC^I73T^GATA1^KO^	5.73 ± 1.22	6.57 ± 1.87	7.54 ± 1.78	2.28 ± 0.39^c^
Classical Inflammatory Monocytes CD43^-^CD11c^-^Ly6Chi	SPC^I73T^GATA1^WT^	3.83 ± 1.01	5.37 ± 1.86	2.62 ± 0.48	3.32 ± 0.49
	SPC^I73T^GATA1^KO^	4.49 ± 1.04	5.28 ± 1.40	4.75 ± 1.13	1.34 ± 0.29
Interstitial Macrophages CD43^+^CD11c^+^	SPC^I73T^GATA1^WT^	3.45 ± 0.91	3.91 ± 1.51	0.53 ± 0.12	5.27 ± 0.65^c^
	SPC^I73T^GATA1^KO^	5.80 ± 0.68	1.94 ± 0.77	1.36 ± 0.60	5.15 ± 1.03
Alternative Monocytes CD43^+^CD11c^-^	SPC^I73T^GATA1^WT^	0.71 ± 0.20	0.74 ± 0.40	0.06 ± 0.02	1.33 ± 0.14^c^
	SPC^I73T^GATA1^KO^	0.40 ± 0.05	0.42 ± 0.13	0.30 ± 0.19	0.77 ± 0.15
Monocyte-derived Macrophages CD43^-^CD11c^+^	SPC^I73T^GATA1^WT^	1.44 ± 0.42	2.52 ± 0.94	2.67 ± 0.17	1.85 ± 0.11
	SPC^I73T^GATA1^KO^	1.37 ± 0.25	2.57 ± 0.69	3.94 ± 0.51^a^	1.44 ± 0.18^c^
Monocyte-derived Macrophages CD206^-^CD43^-^CD11c^+^	SPC^I73T^GATA1^WT^	0.53 ± 0.09	0.98 ± 0.34	0.87 ± 0.10	0.41 ± 0.04
	SPC^I73T^GATA1^KO^	0.78 ± 0.15	0.88 ± 0.25	1.07 ± 0.15	0.33 ± 0.06
Monocyte-derived Macrophages CD206^+^CD43^-^CD11c^+^	SPC^I73T^GATA1^WT^	0.91 ± 0.34	1.54 ± 0.59	1.80 ± 0.08	1.44 ± 0.15
	SPC^I73T^GATA1^KO^	0.59 ± 0.17	1.69 ± 0.47	2.86 ± 0.63^a^	1.11 ± 0.13^c^

Single cell suspensions of SP-C^I73T^GATA1^WT^ and SP-C^I73T^GATA1^KO^ lung digests was assessed by flow cytometry, enriched in viable CD45^+^ singlets, and analyzed following gating strategy described in the *Methods* section. Relative abundance (%) is presented as means ± SEM (N=4-6 mice/condition). Data were analyzed by two-way ANOVA, with Tukey post-hoc test.

a Significant compared to controls (CTL).

b Significant compared to 7 d group.

c Significant compared to14 d group.

**TABLE 3:** Lymphoid populations (absolute counts) in the lung of SP-C^I73T^GATA1^WT^ and SP-C^I73T^GATA1^KO^ mice.

Lymphoid	Genotype	CTL *(n=6)*	7d *(n=5)*	14d *(n=4)*	42d *(n=4)*
Total Lymphocytes CD3^+^	SPC^I73T^GATA1^WT^	345,631 ± 86,296	183,554 ± 39,269	395,329 ± 56,594	190,527 ± 65,449
	SPC^I73T^GATA1^KO^	250,269 ± 79,960	360,842 ± 47,500	589,159 ± 253,538	126,307 ± 25,065^c^
T Cells CD3^+^CD4^+^	SPC^I73T^GATA1^WT^	195,017 ± 54,159	69,888 ± 14,055	143,506 ± 17,513	82,764 ± 28,488
	SPC^I73T^GATA1^KO^	96,382 ± 29,041	160,060 ± 22,763	261,151 ± 119,068	64,875 ± 12,762
T Cells CD3^+^CD8^+^	SPC^I73T^GATA1^WT^	56,669 ± 14,499	42,242 ± 10,950	89,201 ± 7,790	34,887 ± 12,703
	SPC^I73T^GATA1^KO^	81,096 ± 27,228	75,407 ± 1,942	146,561 ± 78,064	18,951 ± 4,028^c^
Dendritic Cells CD4^+^CD103^+^	SPC^I73T^GATA1^WT^	5,220 ± 1,415	4,584 ± 1,254	61,332 ± 22,982^a^ ^,^ ^b^	6,324 ± 2,890^c^
	SPC^I73T^GATA1^KO^	1,894 ± 549	20,495 ± 10,423	25,963 ± 8,265	4,703 ± 722
Dendritic Cells CD8^+^CD103^+^	SPC^I73T^GATA1^WT^	63,210 ± 14,376	46,197 ± 13,312	24,085 ± 2,449	42,933 ± 14,522
	SPC^I73T^GATA1^KO^	39,530 ± 15,995	66,423 ± 7,023	55,100 ± 30,155	20,219 ± 4,546
Total B Cells B220^+^	SPC^I73T^GATA1^WT^	355,729 ± 116,677	121,006 ± 36,745	174,755 ± 42,299	185,453 ± 74,308
	SPC^I73T^GATA1^KO^	575,678 ± 176,291	277,739 ± 66,428	380,566 ± 195,524	79,770 ± 17,796^a^
B Cells B220^+^CX3CR1^-^	SPC^I73T^GATA1^WT^	136,526 ± 50,823	34,278 ± 12,104	105,394 ± 18,429	79,686 ± 33,316
	SPC^I73T^GATA1^KO^	161,705 ± 47,002	105,698 ± 40,083	75,482 ± 42,431	10,523 ± 2,139^a^
B Cells B220^+^CX3CR1^+^	SPC^I73T^GATA1^WT^	266,498 ± 75,310	86,729 ± 24,875	69,362 ± 26,868	105,766 ± 41,078
	SPC^I73T^GATA1^KO^	413,972 ± 129,402	172,040 ± 27,728	262,333 ± 170,944	69,247 ± 15,758^a^

Single cell suspensions of SP-C^I73T^GATA1^WT^ and SP-C^I73T^GATA1^KO^ lung digests was assessed by flow cytometry, enriched in viable CD45^+^ singlets, and analyzed following gating strategy described in the *Methods* section. Absolute counts are presented as means ± SEM (N=4-6 mice/condition). Data were analyzed by two-way ANOVA, with Tukey post-hoc test.

^a^Significant compared to controls (CTL).

^b^Significant compared to 7 d group.

^c^Significant compared to 14 d group.

**TABLE 4:** Lymphoid populations (relative abundance) in the lung of SP-C^I73T^GATA1^WT^ and SP-C^I73T^GATA1^KO^ mice.

Lymphoid	Genotype	CTL *(n=6)*	7d *(n=5)*	14d *(n=4)*	42d *(n=4)*
Total Lymphocytes CD3^+^	SPC^I73T^GATA1^WT^	17.33 ± 1.36	9.71 ± 2.54^a^	6.43 ± 1.51^a^	20.97 ± 1.14^b^ ^,^ ^c^
	SPC^I73T^GATA1^KO^	12.51 ± 0.85	14.58 ± 3.08	15.27 ± 1.56	21.32 ± 1.26^a^
T Cells CD3^+^CD4^+^	SPC^I73T^GATA1^WT^	9.50 ± 0.60	3.64 ± 0.87^a^	2.29 ± 0.42^a^	9.20 ± 0.55^b^ ^,^ ^c^
	SPC^I73T^GATA1^KO^	4.96 ± 0.44	6.51 ± 1.48	7.90 ± 1.42	10.98 ± 0.91^a^ ^,^ ^b^
T Cells CD3^+^CD8^+^	SPC^I73T^GATA1^WT^	0.89 ± 0.16	1.10 ± 0.28	0.22 ± 0.03	3.05 ± 1.20^a^ ^,^ ^c^
	SPC^I73T^GATA1^KO^	1.77 ± 0.20	1.48 ± 0.23	0.85 ± 0.27	1.64 ± 0.17
Dendritic Cells CD4^+^CD103^+^	SPC^I73T^GATA1^WT^	0.23 ± 0.02	0.21 ± 0.04	0.92 ± 0.40	0.62 ± 0.14
	SPC^I73T^GATA1^KO^	0.10 ± 0.01	0.81 ± 0.41	0.73 ± 0.14	0.84 ± 0.07
Dendritic Cells CD8^+^CD103^+^	SPC^I73T^GATA1^WT^	3.36 ± 0.41	2.41 ± 0.80	0.42 ± 0.11^a^	4.78 ± 0.09^b^ ^,^ ^c^
	SPC^I73T^GATA1^KO^	1.87 ± 0.32	2.69 ± 0.58	1.28 ± 0.33	3.38 ± 0.39^c^
Total B Cells B220^+^	SPC^I73T^GATA1^WT^	20.06 ± 2.95	7.38 ± 3.07^a^	3.22 ± 1.15^a^	18.64 ± 1.56^b^ ^,^ ^c^
	SPC^I73T^GATA1^KO^	28.35 ± 2.07	12.07 ± 4.08^a^	9.42 ± 1.68^a^	13.67 ± 1.18^a^
B Cells B220^+^CX3CR1^-^	SPC^I73T^GATA1^WT^	8.03 ± 1.60	2.06 ± 0.90^a^	1.93 ± 0.59^a^	7.49 ± 1.01^b^ ^,^ ^c^
	SPC^I73T^GATA1^KO^	8.17 ± 0.49	4.66 ± 1.93	1.71 ± 0.55^a^	1.84 ± 0.17^a^
B Cells B220^+^CX3CR1^+^	SPC^I73T^GATA1^WT^	12.03 ± 2.01	5.32 ± 2.19	1.29 ± 0.63^a^	11.15 ± 0.70^c^
	SPC^I73T^GATA1^KO^	20.18 ± 1.66	7.42 ± 2.27^a^	5.47 ± 2.09^a^	11.83 ± 1.07^a^

Single cell suspensions of SP-C^I73T^GATA1^WT^ and SP-C^I73T^GATA1^KO^ lung digests was assessed by flow cytometry, enriched in viable CD45^+^ singlets, and analyzed following gating strategy described in the *Methods* section. Relative abundance (%) is presented as means ± SEM (N=4-6 mice/condition). Data were analyzed by two-way ANOVA, with Tukey post-hoc test.

a Significant compared to controls (CTL).

b Significant compared to 7 d group.

c Significant compared to14 d group.

**FIGURE 6:**
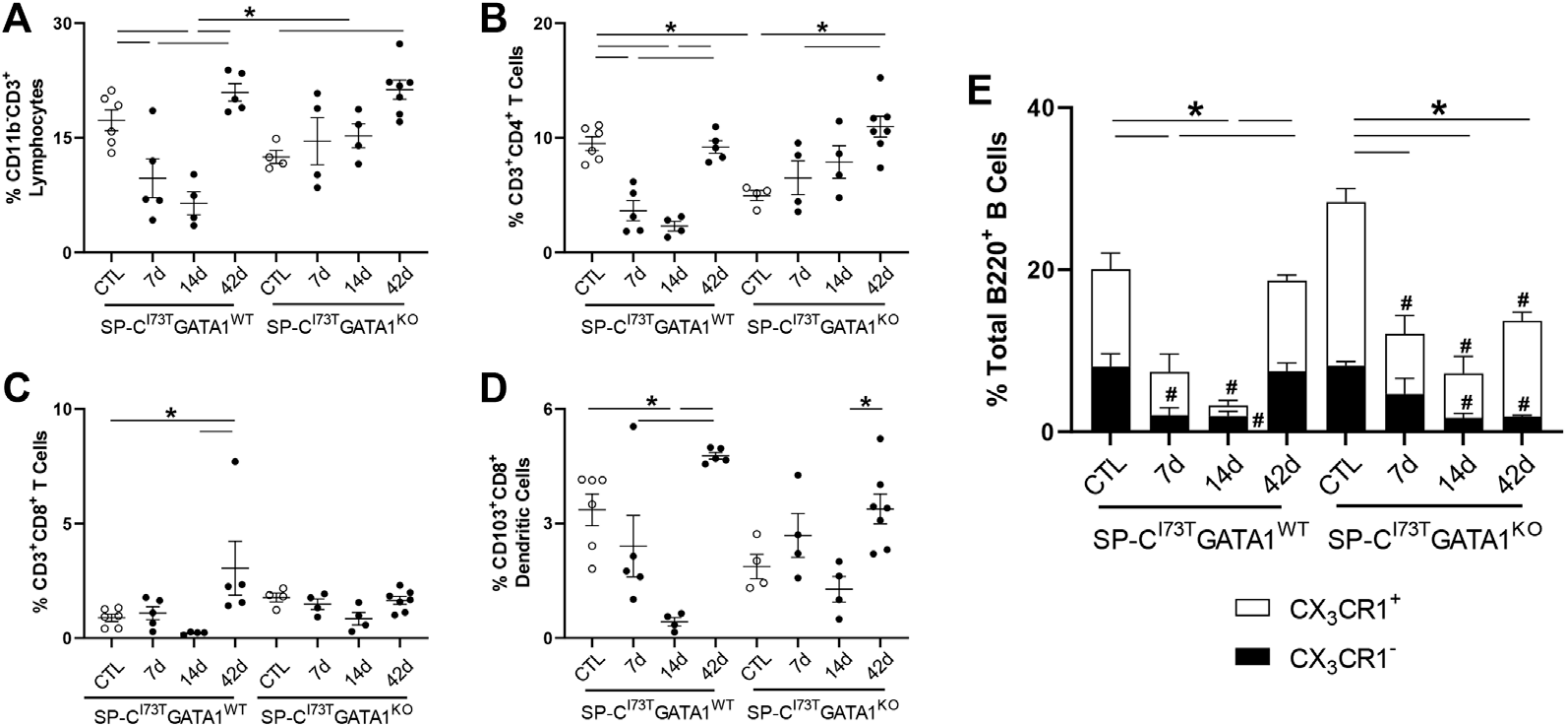
Effects of genetic eosinophil lineage ablation on lymphoid inflammatory cell dynamics following SP-C^I73T^ induced injury. **(A–D)** Flow cytometric quantification of **(A)** CD11b^−^CD3^+^ neutrophils, **(B)** CD3^+^CD4^+^ T cells, **(C)** CD3^+^CD8^+^ T cells, and **(D)** CD103^+^CD8^+^ dendritic cells from collagenase digested lungs from oil treated controls (Ctl), or tamoxifen treated SP-C^I73T^GATA1^WT^ and SP-C^I73T^GATA1^KO^ mice at 7 days, 14 days, or 42 days post injury. A *p* < 0.05 (*) was considered significant. Lines mark significant groups. **(E)** Relative abundance of total of B220^+^ B-cells in SP-C^I73T^GATA1^WT^ and SP-C^I73T^GATA1^KO^ mice. Data are presented as mean ± SEM (*n* = 4–6 mice/group), analyzed using two-way ANOVA. A *p* < 0.05 was considered significant. Note that each column comprises CX_3_CR1^−^ (black); and CX_3_CR1^+^ (white) subsets. *Identifies significant differences in the total population compared to air controls. #Identifies significant differences within CX_3_CR1 subsets versus control groups.

Investigation of the lymphoid compartment showed a drop in total CD11b^−^CD3^+^ lymphocytes in SP-C^I73T^GATA1^WT^ mice 7–14 days post-SP-C^I73T^-induced injury, an effect lessened by eosinophil deletion (Figure 6A). Further analysis revealed reduced CD4^+^ T cells abundance, while the CD8^+^ subset remained unchanged (Figures 6B,C). A comparable time-related change was observed in CD103^+^CD8^+^ dendritic cells identified in both SP-C^I73T^ cohorts (Figure 6D). Histochemical analysis revealed accumulation of CD103^+^ cells predominantly surrounding the blood vessels, with time-related increases in dendritic cells in the lung of SP-C^I73T^GATA1^WT^ cohorts, a pattern reduced in the SP-C^I73T^GATA1^KO^ cohorts (Supplementary Figures S5A,B). In support of our flow cytometry gating, single cell sequencing detected B cells in the lungs of both mouse strains (*Ighd*, Supplementary Figure S1). Relative abundance of total (B220^+^) and CX_3_CR1 expressing B cells were found halved 14 days in both SP-C^I73T^ cohorts (Figure 6E). Decreases in B220^+^ B cells and both CX_3_CR1^-^ subset were noted within 7 days post injury and returned to control levels by 42 days in SP-C^I73T^GATA1^WT^ mice. These changes were prolonged up to 42 days in SP-C^I73T^GATA1^KO^ mice, in particular with respect to the CX_3_CR1^−^ subset. Notably, examination of the absolute counts of these cells showed comparable and significant B cell reduction solely in SP-C^I73T^GATA1^KO^ mice (Supplementary Figure S3B).

### Corticosteroid Treatment Diminishes SP-C^I73T^ Induced Injury Through Eosinophil Inhibition

**FIGURE 7:**
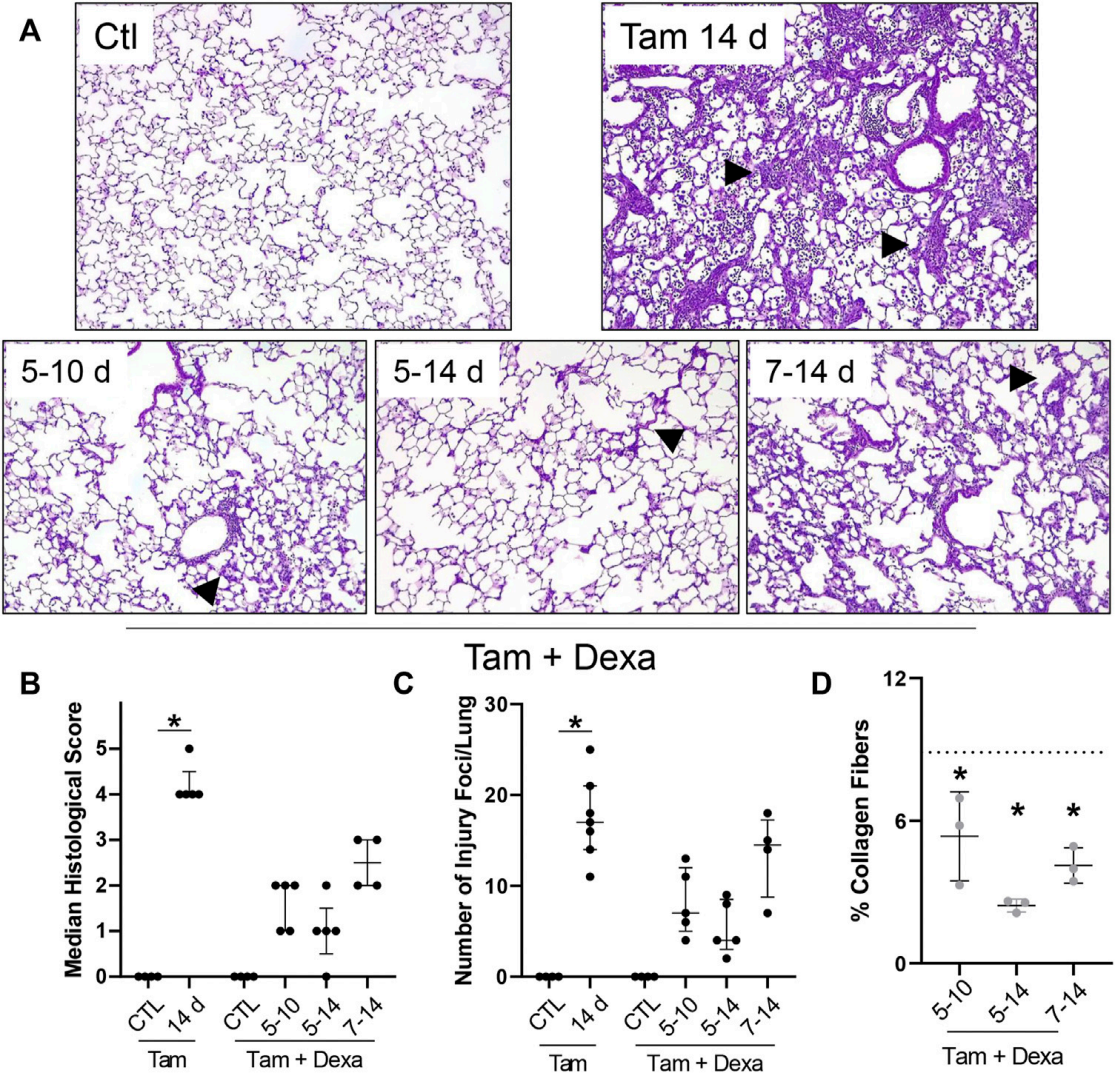
Histopathological effects of dexamethasone administration during SP-C^I73T^ induced injury. **(A)** Hematoxylin and Eosin stain of tissue sections from control (Ctl, oil treated SP-C^I73T^ mice), tamoxifen treated, or tamoxifen + dexamethasone treated SP-C^I73T^ mice 14 days after injury. Three dexamethasone groups are described (1 mg/kg), receiving daily i.p. injections between day 5–10, 5–14, or 7–14 following tamoxifen induction. Arrowheads represent regions of interest, including septal remodeling edema, cell infiltrate. Original magnification: ×100. Representative images from at least 3 mice/group are shown. **(B,C)** Histological scoring and quantification of foci of injury, shown as Median ± interquartile range, of control (Ctl, oil treated SP-C^I73T^ mice), tamoxifen treated, or tamoxifen + dexamethasone treated (day 5–10; 5–14; 7–14) SP-C^I73T^ mice 14 days after injury. **(D)** Quantification of collagen fiber content in tamoxifen + dexamethasone treated SP-C^I73T^ mice 14 days after injury. Three dexamethasone groups are described (1 mg/kg), receiving daily i.p. injections between day 5–10, 5–14, or 7–14 following tamoxifen induction. Each point represents the mean of five ×100 images taken from each section, converted to 8-bit on ImageJ, and relative amount measured using constant threshold set to produce between 1 and 2% collagen in control tissues. Of note, all images avoided large airways and blood vessels. Images for tamoxifen treated groups were selected from foci of injury. Data are presented as mean ± SD (*n* = 3–4), analyzed using two-way ANOVA. *represents groups significantly different (*p* < 0.05) compared to was collagen level in tamoxifen treated specimens (shown as a dotted line).

**FIGURE 8:**
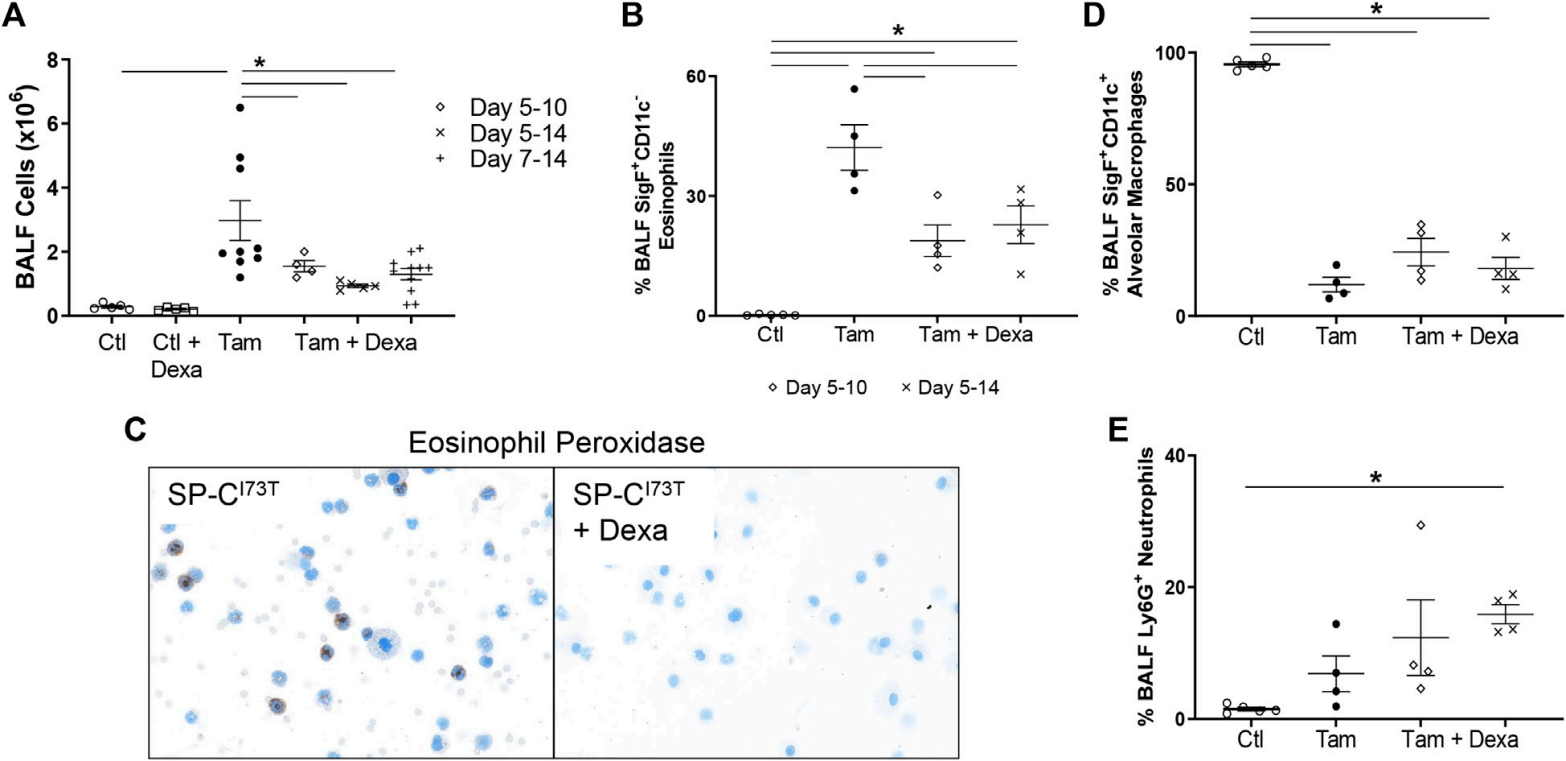
Inflammatory cell changes in dexamethasone treated mice during SP-C^I73T^ induced injury. **(A)** Bronchoalveolar lavage fluid (BAL) cell counts from control (CTL, oil and dexamethasone treated SP-C^I73T^ mice) or SP-C^I73T^ mice 14 days post SP-C^I73T^ induced injury. Three dexamethasone groups are described (1 mg/kg), receiving daily i.p. injections between day 5–10, 5–14, or 7–14 following tamoxifen induction. Controls + dexamethasone group received ten daily injections (equivalent to the lengthiest dexamethasone administration). Data are presented as mean ± SEM (*n* = 4–9 mice/group), analyzed using two-way ANOVA. A *p* < 0.05 (*) was considered significant. Lines mark significant groups. **(B)** Flow cytometric analysis of BAL CD11b^+^CD11c^−^SigF^+^ eosinophils from control (Ctl, oil or dexamethasone treated SP-C^I73T^ mice), tamoxifen treated, or tamoxifen + dexamethasone treated SP-C^I73T^ mice 14 days after injury. Two dexamethasone groups are described (1 mg/kg), receiving daily i.p. injections between day 5–10 or 5–14 following tamoxifen induction. Data presented as mean ± SEM (*n* = 4–5 mice/group), analyzed using one-way ANOVA. A *p* < 0.05 (*) was considered significant. **(C)** Eosinophil Peroxidase staining of BAL cytospins prepared 14 days after tamoxifen (left panel) or tamoxifen + dexamethasone (right panel) treatment. **(D,E)** Flow cytometric analysis of BAL **(D)** CD11b^−^CD11c^+^SigF^+^ alveolar macrophages and **(E)** Ly6G^+^ neutrophils from control (Ctl, oil or dexamethasone treated SP-C^I73T^ mice), tamoxifen treated, or tamoxifen + dexamethasone treated SP-C^I73T^ mice 14 days after injury. Two dexamethasone groups are described (1 mg/kg), receiving daily i.p. injections between day 5–10 or 5–14 following tamoxifen induction. Data presented as mean ± SEM (*n* = 4–5 mice/group), analyzed using one-way ANOVA. A *p* < 0.05 (*) was considered significant.

The first line of therapy against acute inflammatory exacerbations of pulmonary fibrosis is represented by broad-spectrum corticosteroids, including prednisone and dexamethasone (Juarez et al., 2015). Our single-cell sequencing analysis highlighted “Dexamethasone” and “Glucocorticoid” associated signaling in the eosinophil cluster, with a group of genes uniquely downregulated in granulocytes (*Prdx1*, *Rpl13*, *Rpl13a*, *Rpl6*, *Rps8*, *S100a10*) (Supplementary Figure S6A), as well as a signature of highly expressed genes (*Btg2, Id1, Ier3, Il1b, Nfkbia, Cxcl3, Cxcr2, Dusp1, Fos, Mmp9*) (Supplementary Figure S6B). We, therefore, explored the impact of corticosteroid treatment on SP-C^I73T^ induced eosinophilia and injury. For these studies, daily i.p. injections of dexamethasone (1 mg/kg in sterile PBS) were performed following three regimens with termination on day 14: 1) treatment between day 5 and 10 post-injury; 2) treatment between day 5 and 14; and 3) daily administrations between day 7 and 14. Histological analysis showed a significant reduction in the number and size of injury foci present in the lung following dexamethasone administration, with additional protection observed in cohorts receiving earlier treatment (Figures 7A–C). Consistent with this notion, picrosirius red staining of tissue sections revealed reduced collagen deposition in dexamethasone treated cohorts, with the day 5–14 therapeutic regimen producing the largest protection (Figure 7D). Cell counts of BAL fluid collected at 14 days showed reduced cell influx in the lungs of dexamethasone treated mice (Figure 8A). Flow cytometric analysis of BAL cells collected from mice receiving tamoxifen or tamoxifen + dexamethasone (therapy initiation on day 5 post SP-C^I73T^ induced injury) revealed significant drops in SigF^+^CD11c^−^ eosinophils, a finding corroborated by cytochemical analysis of BAL cytospins showed reduced expression of eosinophil peroxidase (EPX, Figures 8B,C). By comparison, we did not see changes in SigF^+^CD11c^+^ macrophage abundance following dexamethasone administration (Figure 8D). A significant increase in neutrophilia was noted only in cohorts treated between days 5 and 14 (Figure 8E).

**FIGURE 9:**
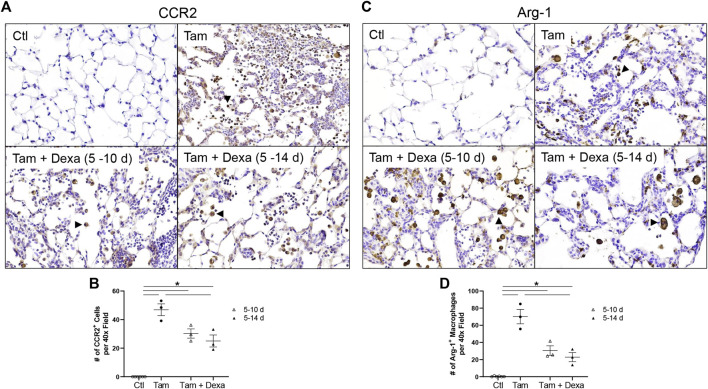
Effects of dexamethasone administration on anti-inflammatory activation and monocyte recruitment during SP-C^I73T^ induced injury. Lung sections immunostained with antibody to **(A)** CCR2 and **(C)** Arg-1 from control (Ctl, oil treated SP-C^I73T^ mice), tamoxifen treated, or tamoxifen + dexamethasone treated SP-C^I73T^ mice 14 days after injury. Two dexamethasone groups are described (1 mg/kg), receiving daily i.p. injections between day 5–10 or 5–14 following tamoxifen induction. Binding was visualized using a Vectastain kit. Arrowheads indicate cells expressing the receptor. Original magnification, ×400. Representative sections from 3 mice/group are shown. Quantification of each respective antibody is shown in **(B,D)** as the average number of brown/positive inflammatory cells in ×400 images of regions of injury. Data are presented as mean ± SD (*n* = 3 mice/group), analyzed using two-way ANOVA. A *p* < 0.05 (*) was considered significant.

Immunohistochemical analysis was used to examine changes in monocyte recruitment (CCR2) and lung inflammatory cell activation (Arg-1) following dexamethasone treatment. We found increased numbers of CCR2^+^ cells in SP-C^I73T^ mice, that was progressively reduced by dexamethasone administration (Figures 9A,B). By comparison, Arg-1 expression was not altered by steroid administration (Figures 9C,D).

## Discussion

The inability of the lung epithelium, in particular a dysfunctional one, to cope with stress is at the root of fibrosis, asthma, and COPD (Spella et al., 2017). These chronic respiratory pathologies are accompanied by a breakdown in communication with the immune compartment. When left unsupervised inflammatory cells promote the amplification of injury signals, leading to irreversible loss of function and scarring (Hambly et al., 2017). Pulmonary fibrosis was long thought to produce a steady decline in pulmonary function, a view ratified by the existence of acute exacerbations, highly inflammatory events that significantly influence morbidity and mortality of patients. Because of their complex temporal, spatial and phenotypic heterogeneity, acute inflammatory exacerbations (AIEs) of COPD, asthma, and pulmonary fibrosis are not well characterized or effectively countered with broad-spectrum immunomodulation (corticosteroids, cytokine modulation, anti-TGFβ1) as the current first line of treatment available (Richeldi et al., 2003; Papiris et al., 2012; Behr and Richeldi, 2013). Improvements in COPD and asthma therapy can be attributed, at least in part, to the classification of exacerbation endotypes, enriched in either monocytes, neutrophils, or eosinophils (Magnini et al., 2017; Nakagome and Nagata, 2018; Yun et al., 2018; Barnes, 2019; Hoffmann-Vold et al., 2019; Kuruvilla et al., 2019). In this study we took under examination the eosinophilic endotype in PF exacerbation (Libby, 1987; Yousem, 2000; Brix et al., 2016; Kakugawa et al., 2016). Here we show that inflammatory exacerbations triggered by a PF-linked mutation of the surfactant protein C (SP-C^I73T^) produce an eosinophilic phenotype. Single-cell RNA sequencing profiling of these cells identified genetic signatures associated with inflammation and proliferation (MYC, YAP, STAT3, and IL-6 signaling). Deletion of eosinophil lineage resulted in improved survival and reduced inflammatory cell influx throughout the injury course, while pharmacological intervention with corticosteroids effectively reduced eosinophilic burden and improved disease outcome.

Prior experimental modeling of pulmonary fibrosis has relied heavily on chemical induced injury (e.g., nitrogen mustard, bleomycin, silica) to cause direct damage to structural epithelium and resident immune cells, progressing to tissue remodeling (Venosa et al., 2016; Liu et al., 2017; Tashiro et al., 2017). Our group and others have identified monocytes and monocytes-derived moieties as a major population undergoing early expansion during chemical induced fibrogenic responses (Venosa et al., 2016; Misharin et al., 2017; Aran et al., 2019; Reyfman et al., 2019; Joshi et al., 2020). We have also shown that monocyte/macrophage moieties play important role in the initial stages of acute exacerbations (Venosa et al., 2019; Venosa et al., 2021). The clinical paucity of effective targeted monocyte/macrophage anti-fibrotic therapy suggests that there may be additional key players in the tissue remodeling, particularly in those populations where intrinsic epithelial stress (mutations) drives injury, rather than exogenous triggers. Thus, implementation of genetic models of spontaneous injury and remodeling that more closely reflect human disease allows us to advance our understanding of lung fibrosis. Mutations in the *SFTPC* gene have been linked to varying degrees of fibrotic disease in adults and pediatric patients (Abou Taam et al., 2009; Crossno et al., 2010; Beers et al., 2011). Guided by epidemiological evidence, murine expression of the isoleucine to threonine substitution at position 73 in the SFTPC generates parenchymal injury consistent with a highly eosinophilic acute exacerbation (Brasch et al., 2004; Hawkins et al., 2015; Nureki et al., 2018). We initially performed single-cell sequencing analysis of digested lung tissues, to unbiasedly identify and compare the phenotypes of all immune populations accumulating at the peak of SP-C^I73T^ induced inflammation. Our pathway analysis identifies signatures related to survival (mTOR, MYC, YAP) and pro-inflammatory activation (IL-6, IL-17, IL-4/13). Survival pathways have been previously shown to be linked to eosinophil persistence in the tissue in inflammatory allergic conditions (Zhu et al., 2018). Eosinophil expression of inflammatory mediators is well described in the context of asthma and infection (Piehler et al., 2011; Doran et al., 2017). Furthermore, our findings that “Dexamethasone” and “Corticosteroid” signaling are highly regulated during the peak of the SP-C^I73T^ induced inflammatory exacerbation represented the foundation of our subsequent studies on the efficacy of steroid therapy. Our histological, histochemical, and flow cytometric analyses all support the notion that dexamethasone is effective in reducing eosinophilia without impacting the numbers of CCR2^+^ monocyte accumulation in the lung, resulting in beneficial effects on the trajectory of the disease. This observation is in line with evidence that eosinophilic exacerbations of asthma and COPD are responsive to steroid therapy (Barnes et al., 2016; Cheng, 2018; Nakagome and Nagata, 2018; Barnes, 2019; Morantes-Caballero and Fajardo Rodriguez, 2019; Chipps et al., 2020). Of note is our findings that tissue protection depended on the time of initiation and therapy duration, with earlier initiation (day 5 post tamoxifen) associated with a better outcome. Our previously published findings that by day 7 the SP-C^I73T^ lung is moderately eosinophilic may help justify this observation (Nureki et al., 2018). We did take into consideration extending dexamethasone therapy throughout the 42 days fibrotic cycle induced by mutant SP-C, as well as the termination of therapy shortly after the peak of inflammation followed analysis of lung structure at 42 days. However, problems related to stress induced by several weeks of daily i.p. injections and the potential impact of hormonal replacement on adrenal function obstructed our ability to perform these complementary studies. Transition to a minipump for the controlled delivery of dexamethasone, as well as optimization of a drug-tapering strategy, is currently being evaluated. Together, these results indicate that corticosteroid efficacy, which is clinically variable, may be dependent on the endotype of the disease, a notion consistent with observations made in COPD and asthma (Cheng, 2018; Chipps et al., 2020).

Monocytes and macrophages represent the central players in the inflammatory phase of fibrogenesis, particularly that induced by chemical exposure (e.g., bleomycin and silica) (Laskin et al., 2019; Mukherjee et al., 2021; Golden et al., 2022). This idea is driven by substantial evidence that depletion of phagocytic cells significantly impacts the course of injury, and ultimately fibrogenesis (Misharin et al., 2017; Venosa et al., 2019). In line with this genetic and pharmacological approaches, and in an effort to fill the knowledge gap pertaining to eosinophilic exacerbations of a fibrotic phenotype, we provided evidence that eosinophil lineage deletion is protective against SP-C^I73T^ induced injury. Histological evaluation of GATA1^KO^ cohorts revealed a reduction in injury features and tissue remodeling. One of the reasons for the incomplete phenotypic rescue is due to the residual stress induced by monocytic and neutrophilic inflammation following SP-C^I73T^ induction. More dramatic were the results related to BAL inflammation measured at the peak of exacerbation (14 days), a time canonically associated with eosinophilia. In line with these results, flow cytometric analysis of lung tissue from SP-C^I73T^GATA1^WT^ and SP-C^I73T^GATA1^KO^ mice show seemingly comparable immune cell proportions up to 7 days post-injury. By comparison, findings of prolonged neutrophilia in the SP-C^I73T^GATA1^KO^ groups up to 14 days may represent a compensatory response arising to overcome eosinophil deletion. Similarly, BAL cytokine expression analysis shows increased expression for the monocyte recruitment factor, *mcp1,* in the knockout mice. Notably, there was no change in classical inflammatory monocyte influx between the two cohorts at 14 days, likely because these cells were previously shown to infiltrate the lung at earlier stages of the injury (Venosa et al., 2019). Monocyte-derived moieties, gated on CD64 high, CD11c high, SiglecF low expression was recently linked to pro-fibrotic remodeling following blemycin-induced lung injury (Misharin et al., 2017; Joshi et al., 2020). Our characterization split them by their expression for the mannose receptor, CD206. In particular, we observed increases in total and CD206^+^ monocyte-derived macrophages in SP-C^I73T^GATA1^KO^ mice. While not definitive, initial *in situ* hybridization analysis of *tgfb1* in CD206 expressing cells show double-positive cells in the SP-C^I73T^GATA1^KO^ mice. A thorough investigation of the phenotype of these CD206^+^ monocyte-derived macrophages is currently being undertaken.

In addition to the analysis of myeloid populations, we examined T and B lymphoid subsets, which have been clinically shown to participate in fibrotic disease (Habiel et al., 2019; Ali et al., 2020). Notably, lymphocyte deletion in mice (e.g., SCID, Rag^KO^) does not significantly alter the fibrotic phenotype after bleomycin or silica exposure, suggesting that T lymphocytes are not necessary for the development of pulmonary fibrosis (Helene et al., 1999; Beamer et al., 2010). This is consistent with our results of a drop in absolute and relative abundance for CD3^+^, CD4^+^, CD8^+^ and dendritic cell populations in the acute phase of an exacerbation, regardless of the genotype. The observation that total B220^+^ cells were significantly reduced in both murine strains remains unexplained. The significance of this observation remains valuable since there is evidence, though sporadic, linking B cells and pulmonary fibrosis (Hoyne et al., 2017; Roman and Chiba, 2021). Indeed, clinical and experimental analysis shows decreases in regulatory B cells, combined with increases in IgA^+^ memory B cells and plasmablasts were found in the bloodand lungs during fibrogenic remodeling (Schiller et al., 2017; Asai et al., 2019). Further research is necessary to elucidate the exact composition and phenotype of B cell subsets (e.g., plasma cells abundance, immunoglobulin repertoire over time, memory phenotype) during an inflammatory exacerbation induced by mutant SP-C^I73T^. Such findings could have repercussions with respect to exacerbation incidence as a result of loss of self-tolerance to lung-specific proteins; aberrant response to infections, a noteworthy co-morbidity factor in pulmonary fibrosis patients; and even responsiveness to standard of care drugs including pirfenidone (Nalysnyk et al., 2012; Juarez et al., 2015; Collard et al., 2016; Karman et al., 2021).

To conclude, this work utilizes the SP-C^I73T^ model of exacerbations to define the phenotype of peripheral eosinophils in pulmonary fibrosis. In addition, we characterize the benefits of eosinophil lineage deletion and standard corticosteroid therapy in eosinophilic AIEs. Notably, flow cytometric analysis also identifies significant changes in monocyte-derived macrophages and B cells at the peak of inflammation, an observation with potential clinical significance in terms of therapy and longitudinal examination of pulmonary fibrosis cohorts. Taken together, this work highlights the importance of modeling specific disease endotypes that could lead to therapeutic advances against inflammatory exacerbations of fibrotic injury.

## Data Availability

The datasets presented in this study can be found in online repositories. The names of the repository/repositories and accession number(s) can be found below: https://www.ncbi.nlm.nih.gov/, GSE196657.

## Abbreviations

AIE, acute inflammatory exacerbations; AT2, alveolar type-2 cell; SP-C^I73T^, surfactant protein-C I73T mutant; ΔdblGATA1/GATA1^KO^, GATA1 binding protein one knock out; ILD, interstitial lung disease; IPF/PF, idiopathic pulmonary fibrosis; COPD, chronic obstructive pulmonary disease; FACS, fluorescence-activated cell sorting; CCR2, chemokine receptor-2; BAL/BALF, bronchoalveolar lavage fluid; MoDMs, monocyte-derived macrophages; Int Macs, interstitial macrophages.
