# Supplementary material for: Immunophenotyping of Acute Inflammatory Exacerbations of Lung Injury Driven by Mutant Surfactant Protein-C: A Role for Inflammatory Eosinophils
Source: Front Pharmacol. 2022 Apr 27;13:875887. doi: 10.3389/fphar.2022.875887 (PMC9094740; doi:10.3389/fphar.2022.875887)
Supplement: Supplementary file 1 [file DataSheet1.PDF]

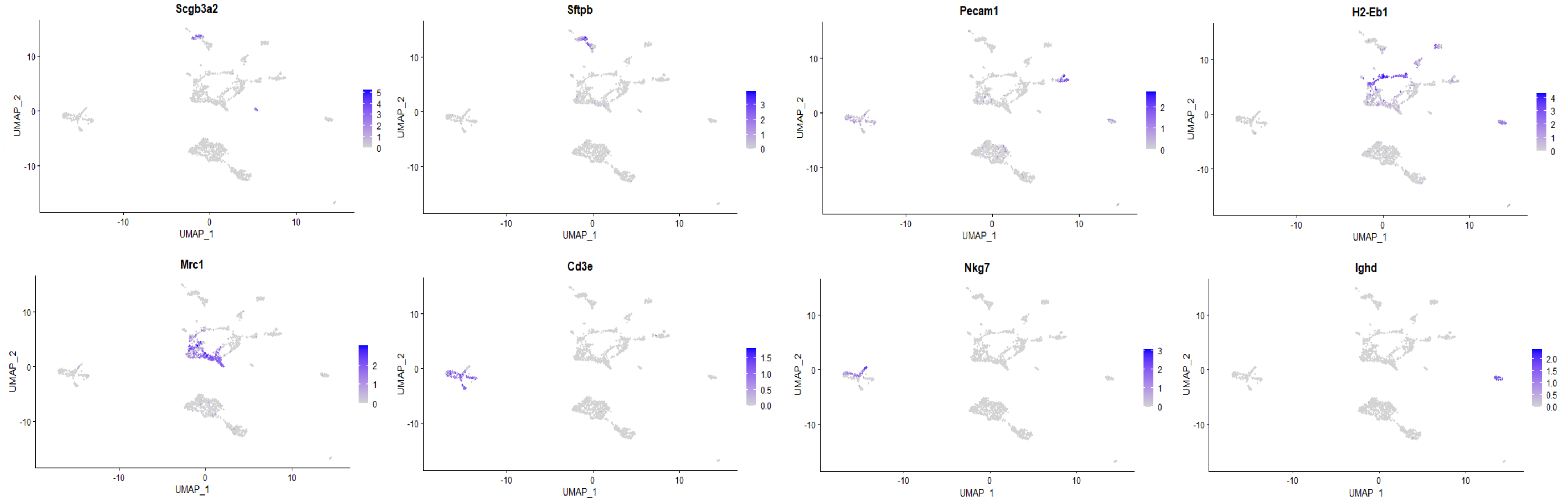

Supp. Fig. 1. Single-cell RNA sequencing analysis of lung tissue digests during SP-C^{I73T} induced acute inflammatory exacerbations. Umaps with overlay of gene expression expressed by epithelial (*Scgb3a2, Sftpb*), endothelial (*Pecam1*), macrophage populations (*H2-Eb1, Mrc1*), T cells (*Cd3e, Nkg7*), or B cells (*Ighd*).

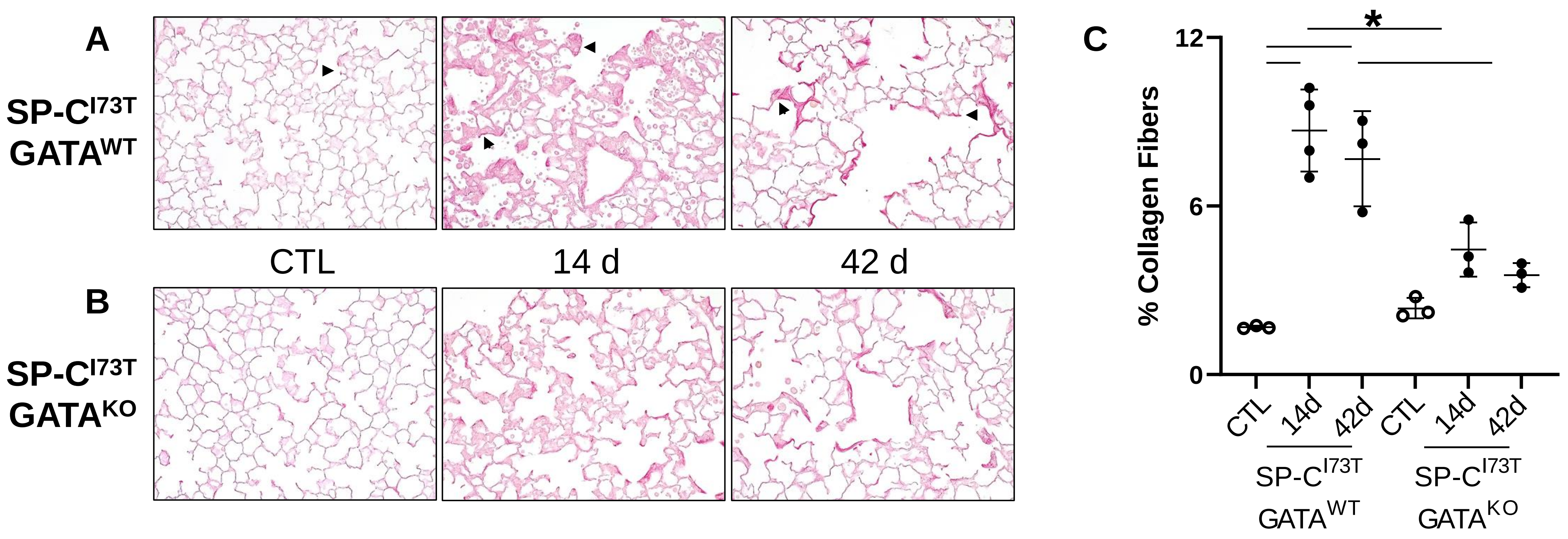

Supp. Fig. 2. Effects of O_3 exposure on collagen SP-C^{I73T}GATA^{WT} and SP-C^{I73T}GATA^{KO} lungs. Representative images of Picrosirius red stained tissue sections from (**A**) SP-C^{I73T}GATA^{WT} and (**B**) SP-C^{I73T}GATA^{KO} mice prepared from control (CTL) mice or 14 - 42 d post tamoxifen induced injury. Arrowheads represents collagen fibers. Original magnification: 100x. Representative images from at least 3 mice/group are shown. Picrosirius Red staining was performed on all samples at once to avoid batch experiment variability. (**C**) Quantification of collagen fiber content. Each point represents the mean of five 100x images taken from each section, converted to 8-bit on ImageJ, and relative amount measured using constant threshold set to produce approximately 1.5% levels in controls. Of note, all images avoided large airways and blood vessels, which contain high levels of collagen fiber. Images for tamoxifen treated groups represented injured areas. Data are presented as mean ± SD (N= 3-4), analyzed using two-way ANOVA. A $p<0.05$ (*) was considered significant.

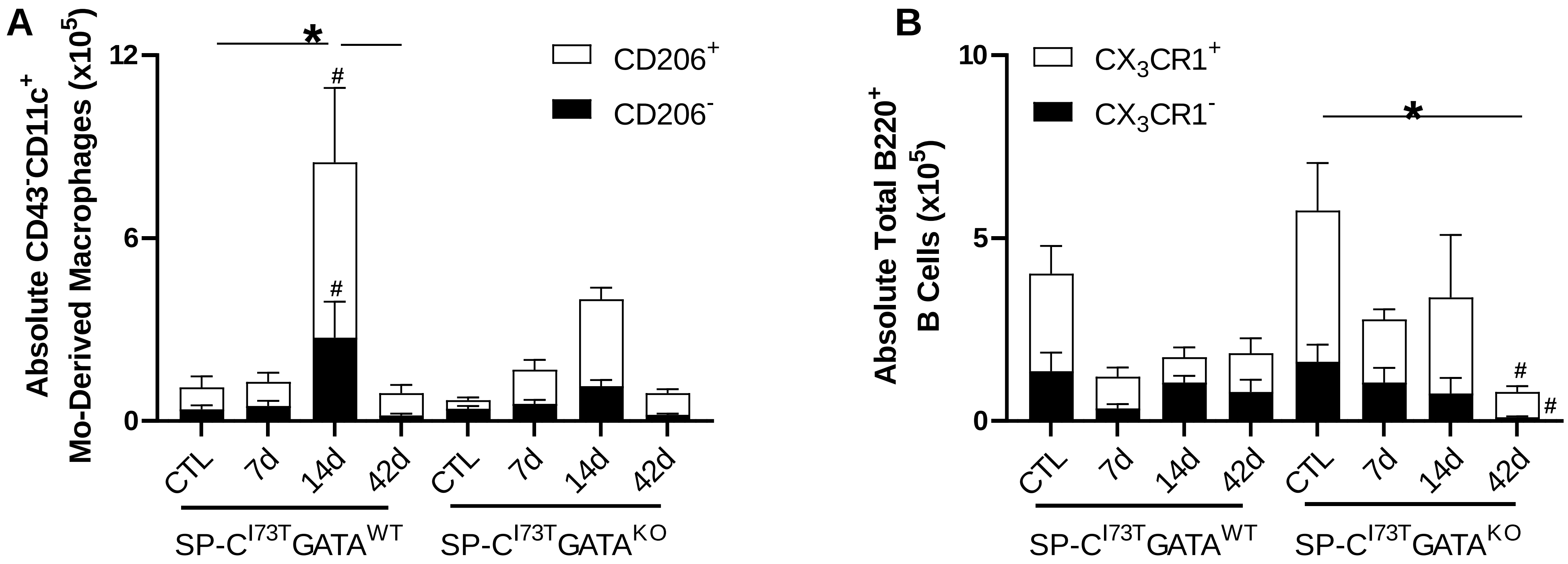

Supp. Fig. 3. Effects of SP-C induced stress on monocyte-derived macrophages and B cell numbers in SP-C^{I73T}GATA^{WT} and SP-C^{I73T}GATA^{KO} mice. Absolute counts of (**A**) CD43^{-}CD11c^{+} monocyte derived macrophages, and (**B**) B220^{+} B cells collected from control (CTL) mice or 7 d, 14 d, 42 d post tamoxifen induced injury. Data are presented as mean ± SE (n = 5-8 mice/group), analyzed using two-way ANOVA. A $p<0.05$ was considered significant. Note that each column includes CD206^{-} or CX_3CR1^{-} (black); and CD206^{+} or CX_3CR1^{+} (white) subsets. ***** Identifies significant differences in the total population compared to air controls. # Identifies significant differences within specific CD206 and CX_3CR1 subsets.

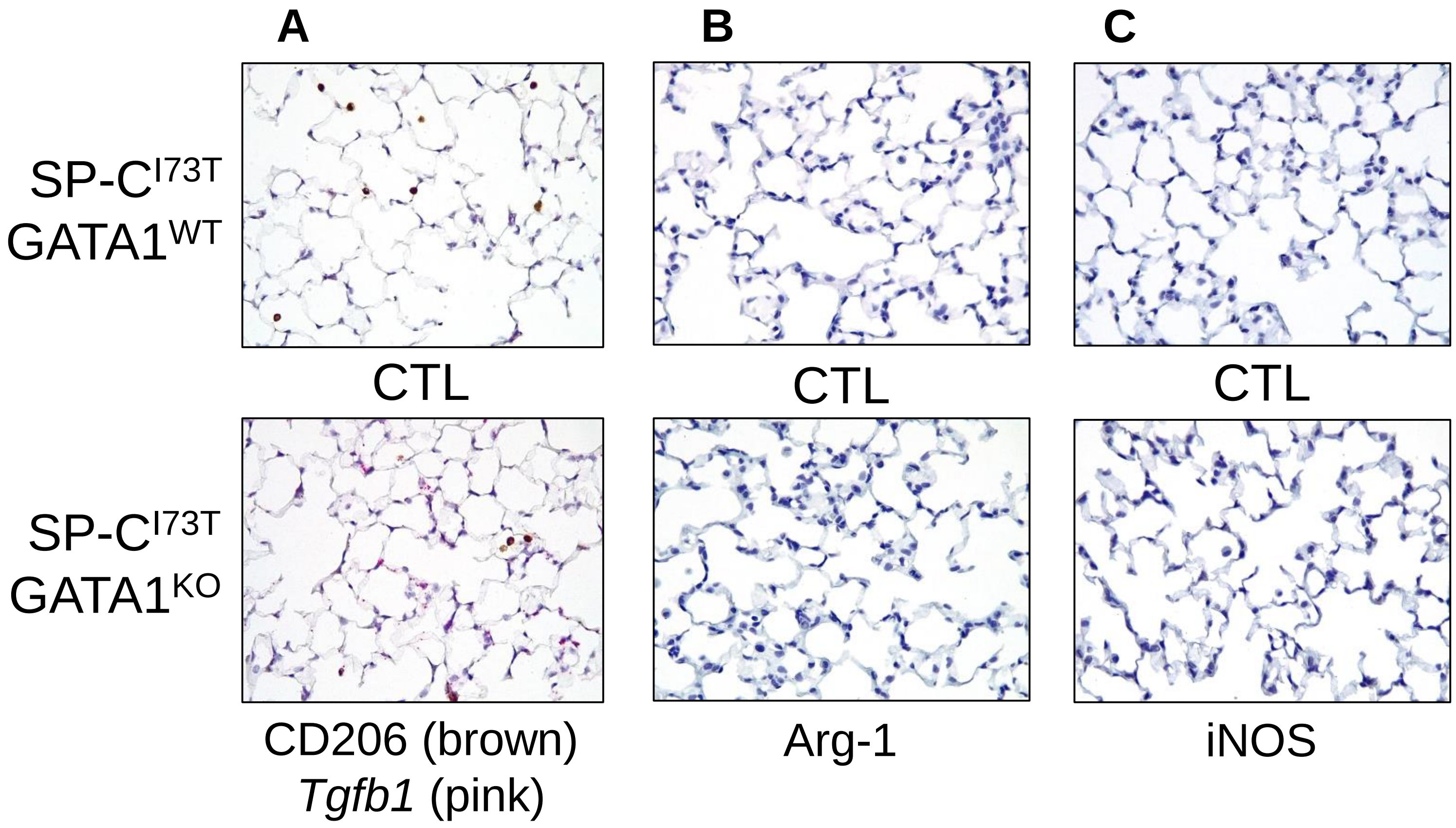

Supp. Figure 4. Effects of genetic eosinophil lineage ablation on cellular activation following SP-C^{I73T} induced injury. Histochemical analysis alone or in combination with in situ hybridization of SP-C^{I73T}GATA1^{WT} and SP-C^{I73T}GATA1^{KO} lungs in control conditions. Sections were immunostained with antibody to (**A**) CD206 + *Tgfb1 (in situ* hybridization*)*. mRNA visualization is shown in pink. Protein expression was visualized using a DAB Vectastain kit (brown). Arrowheads indicate cells expressing the receptor. (**B**) Arg-1 or (**C**) iNOS. Binding was visualized using a Vectastain kit. Original magnification, 400x. Representative region is shown (N = 3).

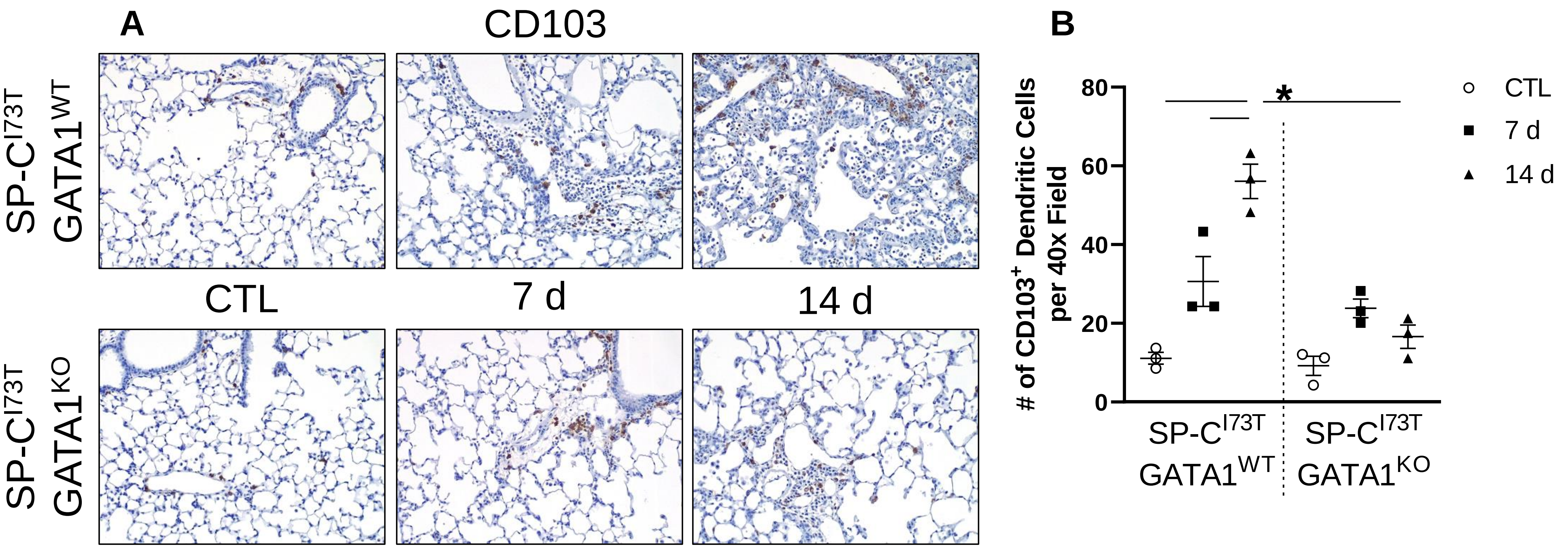

Supp. Figure 5. Effects of genetic eosinophil lineage ablation on dendritic cells following SP-C^{I73T} induced injury. Histochemical analysis alone of SP-C^{I73T}GATA1^{WT} and SP-C^{I73T}GATA1^{KO} lungs from control (CTL) mice or 7 d, 14 d, 42 d post tamoxifen induced injury. Sections were immunostained with antibody to (**A**) CD103. Expression was visualized using a DAB Vectastain kit (brown). Representative images from at least 3 mice/group are shown. Histochemical staining was performed on all samples at once to avoid batch experiment variability. (**B**) Staining quantification calculated as number of CD103^{+} cells per 40x field. Data is represented in mean ± SEM (N = 3). * Indicates significant differences between group ($p < 0.05$).

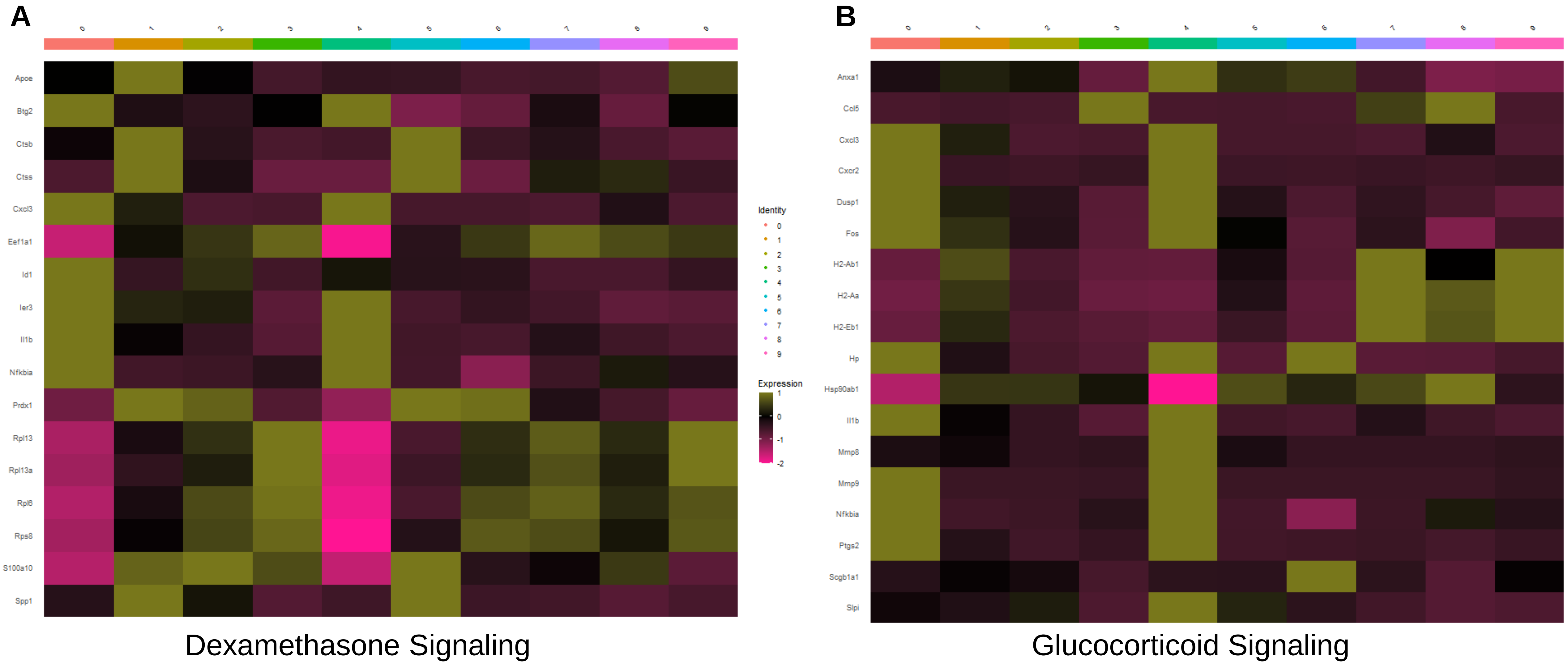

Supp. Figure 6. Dexamethasone and Glucocorticoid associated gene expression in SP-C^{I73T} induced injury. Differentially expressed genes in all 9 annotated clusters isolated from SP-C^{I73T} mice 14 d after injury. Heat-map showing expression of significantly regulated genes involved in (**A**) ‘Dexamethasone Signaling’ and (**B**) ‘Corticosteroid Signaling’ using IPA. A fold change > 1.5 and false discovery rate [q-value] < 0.05 was considered significant. Note signature of genes uniquely expressed in cluster 0 and 4 (eosinophil and neutrophil, respectively) 14 d after SP-C^{I73T} injury.
